# A novel role of exostosin glycosyltransferase 2 (EXT2) in glioblastoma cell metabolism, radiosensitivity and ferroptosis

**DOI:** 10.1038/s41418-025-01503-w

**Published:** 2025-04-15

**Authors:** Rocío Matesanz-Sánchez, Mirko Peitzsch, Inga Lange, Jovan Mircetic, Michael Seifert, Nils Cordes, Anne Vehlow

**Affiliations:** 1 OncoRay – National Center for Radiation Research in Oncology, Faculty of Medicine Carl Gustav Carus, Technische Universität Dresden, Dresden, 01307 Germany; 2 Institute of Clinical Chemistry and Laboratory Medicine, University Hospital Carl Gustav Carus, Technische Universität, Dresden, 01307 Germany; 3 Helmholtz-Zentrum Dresden - Rossendorf, Institute of Radiooncology - OncoRay, Dresden, 01328 Germany; 4 German Cancer Consortium (DKTK), Partner Site Dresden, and German Cancer Research Center (DKFZ), Heidelberg, 69192 Germany; 5 Institute for Medical Informatics and Biometry (IMB), Faculty of Medicine Carl Gustav Carus, Technische Universität Dresden, Dresden, 01307 Germany; 6 Department of Radiotherapy and Radiation Oncology, University Hospital Carl Gustav Carus, Technische Universität Dresden, Dresden, 01307 Germany

**Keywords:** Cancer metabolism, CNS cancer

## Abstract

Glioblastoma (GBM) employs various strategies to resist therapy, resulting in poor patient survival. A key aspect of its survival mechanisms lies in metabolic regulation, maintaining rapid growth and evading cell death. Recent studies revealed the connection between therapy resistance and ferroptosis, a lipid peroxidation-dependent cell death mechanism triggered by metabolic dysfunction. Our aim was to identify novel regulators of therapy resistance in GBM cells. We conducted a comprehensive analysis combining RNA-sequencing data from a panel of human GBM cell models and TCGA GBM patient datasets. We focused on the top-12 differentially expressed gene candidates associated with poor survival in GBM patients and performed an RNA interference-mediated screen to uncover the radiochemosensitizing potential of these molecules and their impact on metabolic activity, DNA damage, autophagy, and apoptosis. We identified exostosin glycosyltransferase 2 (EXT2), an enzyme previously described in heparan sulfate biosynthesis, as the most promising candidate. EXT2 depletion elicited reduced cell viability and proliferation as well as radiochemosensitization in various GBM cell models. Mechanistically, we explored EXT2 function by conducting untargeted and targeted metabolomics and detected that EXT2-depleted GBM cells exhibit a differential abundance of metabolites belonging to S-adenosylmethionine (SAM) metabolism. Considering these metabolic changes, we determined lipid peroxidation and found that the diminished antioxidant capacity resulting from decreased levels of metabolites in the transsulfuration pathway induces ferroptosis. Moreover, modifications of specific SAM and transsulfuration metabolism associated enzymes revealed a prosurvival and ferroptosis-reducing function when EXT2 is depleted. Collectively, our results uncover a novel role of EXT2 in GBM cell survival and response to X-ray radiation, which is controlled by modulation of ferroptosis. These findings expand our understanding of how GBM cells respond to radio(chemo)therapy and may contribute to the development of new therapeutic approaches.

## Introduction

Glioblastoma (GBM), characterized by therapy resistance and destructive infiltration, is one of the cancer types with great unmet need. Surgery and concurrent radiochemotherapy with the DNA alkylating agent temozolomide (TMZ) comprise the standard of care. Almost all patients encounter tumor progression with nearly universal mortality and a median survival between 12 and 16 months. Inherent and acquired resistances are the main reasons for limited treatment success [1, 2]. A more in-depth understanding of these resistances could foster the development of novel, molecular-targeted therapies.

In addition to a myriad of intra- and extracellular factors driving therapy resistance, the GBM metabolome and its reprogramming recently emerged as one of the central aspects of resistance. Metabolic maintenance of, for example, reduction-oxidation balance, dysregulated tricarboxylic acid cycle (TCA) cycle, methionine and arginine metabolisms and macromolecular biosynthesis fundamentally ensures survival, proliferation, and migration [3, 4]. While the influence of hypoxia and pH in connection with the Warburg effect, oxidative phosphorylation and TCA cycle on therapy resistance and tumor progression is well known [3, 5–8], numerous metabolic mechanisms and pathways maintain this reprogramming, which are currently being decoded in various cancers, including GBM. It appears that one of the fundamental processes for transcription, pre-mRNA splicing, DNA damage signaling, and immune signaling is arginine methylation [9]. Among others, a family of nine protein arginine methyltransferases and the co-factor S-adenosylmethionine (SAM) are involved in post-translational modifications occurring on histones, RNA binding proteins, and numerous other cellular proteins [9]. SAM´s antagonizing potential of pathological conditions and its enhancing potential for chemotherapeutics have been demonstrated in various cancer types, but not in GBM, and not in the context of irradiation [10–14].

While SAM is reported to play critical roles in apoptosis and autophagy [15], a connection to ferroptosis also appears via methionine metabolism-regulated epigenetic modifications, including an activation of the transsulfuration pathway [16]. Ferroptosis, an iron-dependent form of non-apoptotic-regulated cell death elicited by overwhelming peroxidation of polyunsaturated fatty acids, is dysregulated under diverse pathologic conditions [15, 17–19]. Previous studies have established a link between ferroptosis and the anti-tumor efficacy of radio- and chemotherapy as well as metabolic pathways [20–22]. However, whether the methionine metabolism is also involved in the regulation of ferroptosis sensitivity remains largely unknown.

Among the large number of metabolic enzymes described as crucial regulators of cancer cell survival and resistance, the glycosyltransferase EXT2 is of interest due to its putative tumor suppressor function whose mutation causes the type II form of multiple exostoses, a disease characterized by the formation of osteochondromas [23]. Further, EXT2 and EXT1 mediate the biosynthesis of heparan sulfate important for cell communication and signaling [24].

To address the function of EXT2 in therapy resistance, we focused on GBM and found a novel function of EXT2 in the metabolic response to X-ray irradiation through modulation of SAM levels and ferroptosis induction. These observations shed further light on survival-promoting metabolic pathways in GBM, which may serve as potential novel therapeutic vulnerabilities.

## Results

### Transcriptome analysis of human GBM models aligned with TCGA data sets identifies potential regulators of GBM therapy response

**Fig. 1:**
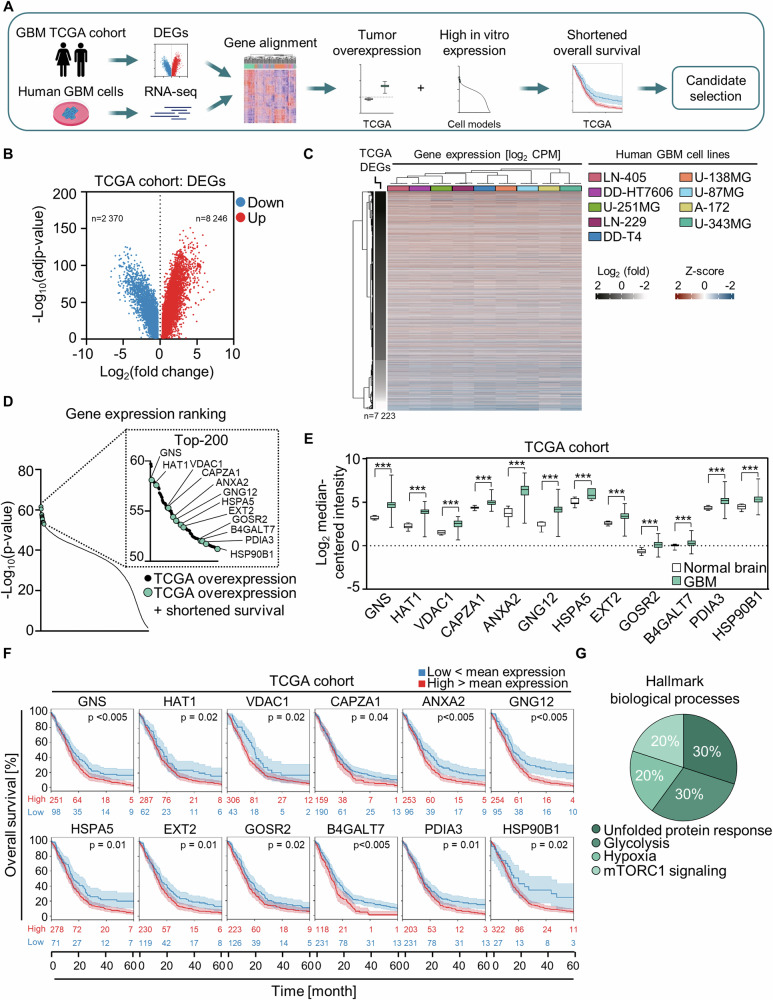
Highly expressed genes prognostic for overall survival of GBM patients fall into the categories unfolded protein response, glycolysis, hypoxia and mTORC1 signaling. **A** Schematic workflow of the GBM transcriptome analysis for candidate identification based on TCGA GBM patient datasets. **B** Depiction of differentially expressed genes (DEG; GBM versus normal brain) found in the TCGA GBM patient cohorts. Significantly up- and downregulated genes are shown in red and blue, respectively, and determined by ANOVA with a cut-off |Log_2_ (fold change GBM versus normal brain)| > 0.3, *p* <  0.05, using GEPIA web tool. **C** Alignment of differential gene expression levels from TCGA, presented as Log_2_ (fold), and the gene expression levels from the RNA-seq data of GBM cell models (Log_2_ CPM; n = 4) shown as Z-score. Heatmap was created with R and data were hierarchically clustered in rows based on DEG and in columns based on gene expression. **D** Waterfall plot of overexpressed genes from TCGA GBM patient cohorts ranked by high gene expression in GBM models displayed as -Log_10_ (*p*-value; *t*-test; *p* < 0.05). The top-200 highly expressed genes were used for the selection of gene candidates associated with shorter overall survival of GBM patients (indicated). **E** Comparative mRNA expression in tumor and normal brain tissue. Data were obtained from TCGA GBM Affymetrix HT HG U133A dataset (Oncomine.com) and analyzed by one-way ANOVA (***p < 0.005). **F** Overall survival analyses based on gene expression (low versus high) from the TCGA GBM patient cohorts. Kaplan-Meier curves display the confidence intervals, the log-rank test *p*-values and the number of patients at risk. Data were downloaded from betastasis.com. **G** Identified hallmark biological processes for the 12 gene candidates from ‘**E**’ and ‘**F**’ using the hallmark gene set collection in gsea-msigdb.org.

We commenced our study by conducting RNA-sequencing in our panel of human GBM models and analyzed TCGA GBM patient datasets to discover novel genes with potential impact on GBM patient survival (Fig. 1A). We focused on genes that are (i) highly expressed in our GBM models, (ii) overexpressed in GBM tumors, and (iii) associate with a shorter overall survival of GBM patients (Fig. 1A–F). Identification of differentially expressed genes (DEG) in TCGA patient cohorts uncovered 8246 higher and 2370 lower expressed genes in GBM relative to normal brain (Fig. 1B). Alignment of the cell model and patient datasets revealed great similarity in the gene expression patterns, with overexpressed genes in GBM tumors (ii; see above) also being highly expressed in our GBM models (i; see above) (Fig. 1C). Filtering based on TCGA GBM overexpressed genes led to a selection of the top-200 significantly highly expressed genes (HEG) in the cell models (Fig. 1D). From these, we identified 12 genes whose overexpression was associated with significant shortening in overall survival of GBM patients (iii; see above) (Fig. 1D–F, Supplementary Fig. S1A, B). Gene enrichment analyses determined an involvement of these genes in hallmark biological processes associated with unfolded protein response, glycolysis, hypoxia, and mechanistic target of rapamycin kinase complex (mTORC1) signaling (Fig. 1G).

### Candidate screening distinguishes EXT2 as key determinant of therapy response in GBM cells

**Fig. 2:**
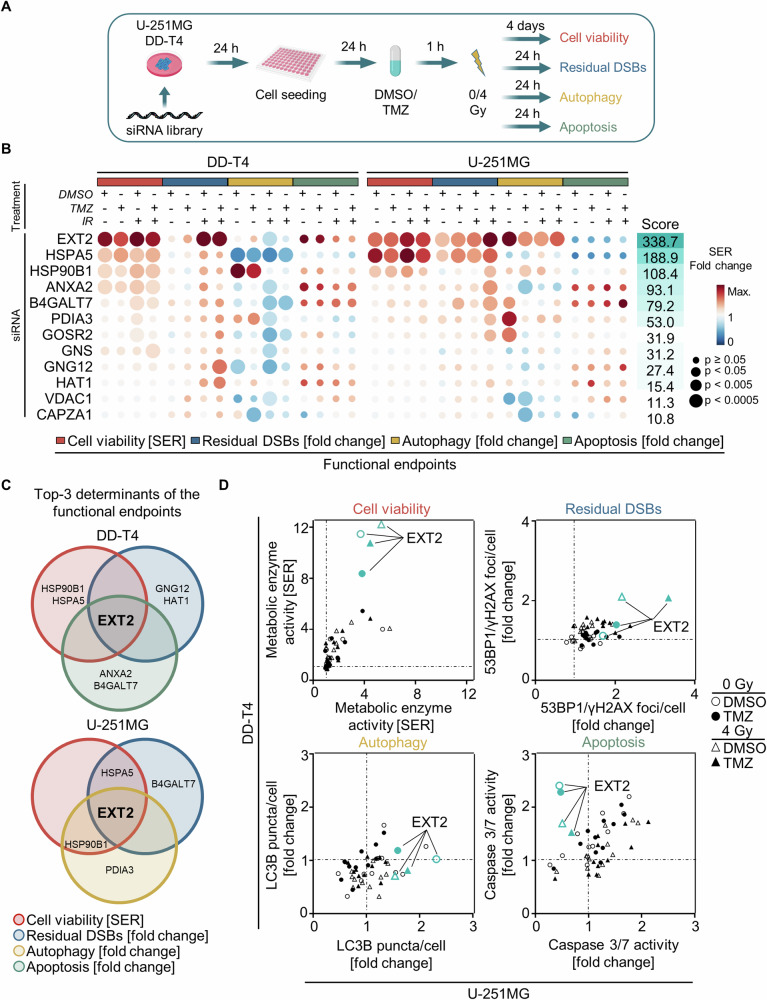
EXT2 emerges as the most potent enhancer of radiochemosensitivity in human GBM models among the 12 identified gene candidates. **A** Workflow of RNAi screen for the 12 candidate genes in combination with temozolomide (TMZ) and X-ray irradiation to evaluate cell viability, residual DSB, autophagy and apoptosis. **B** Heatmap summarizing the effect of each knockdown in indicated treatment groups and for each functional endpoint. Data are visualized in size with p-value and in color scale with either sensitizing enhancement ratio (SER, viability) or fold change (residual DSB, autophagy, apoptosis) relative to controls. The score (-Log_10_ (*p*-value)*SER or fold change) quantifies the knockdown impact of each condition in both cell models. **C** Overlap of the top 3 candidates that decrease (cell viability) or increase (residual DSB, apoptosis, autophagy) the chosen functional endpoints in DD-T4 and U-251MG GBM models. **D** Comparative depiction of indicated readouts (cell viability, residual DSB, autophagy, apoptosis; shown in **B** in TMZ/irradiation exposed DD-T4 and U-251MG cells. Data are shown as mean values normalized to basal conditions and analyzed by one-way ANOVA (*n* = 3).

To assess the role of the 12 genes on major readouts for therapy response, i.e. cell viability, residual DNA double strand breaks (DSB), autophagy, and apoptosis, we conducted a knockdown screen in two GBM models treated with TMZ and X-ray irradiation (Fig. 2A). We found EXT2 eliciting by far the strongest impact on the selected endpoints, also indicated by the calculated summary score of 338.7 for DD-T4 and U-251MG cells (Fig. 2B, Supplementary Fig. S2A–D). Furthermore, EXT2 depletion in combination with X-ray irradiation showed a synergistic effect in reducing the viability of U-251MG cells (Supplementary Fig. S2E). Additional depictions showing the top-3 determinants (Fig. 2C) and a correlation of the observed effects in DD-T4 and U-251MG cells (Fig. 2D) underscore the exceptional role of EXT2 in these GBM models relative to the other genes in regards of the chosen endpoints.

### EXT2 inhibition mediates cytotoxicity, reduced proliferation and radiosensitization in human GBM models

**Fig. 3:**
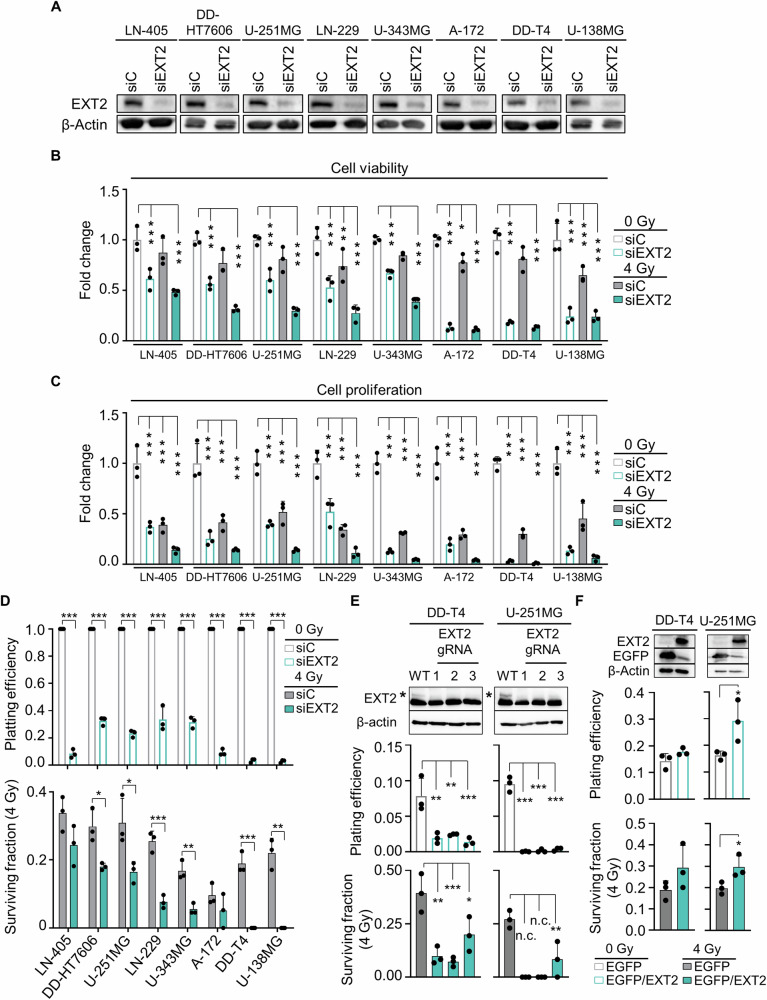
Depletion of EXT2 leads to decreased cell viability and proliferation as well as radiosensitization of human GBM models. **A** Western blotting from whole cell lysates to test for EXT2 knockdown efficacy in indicated GBM models (β-actin served as loading control). Cropped images are displayed. Normalized cell viability (**B**), cell proliferation (**C**), plating efficiency, and clonogenic radiation survival (**D**) of indicated EXT2-depleted GBM models. **E** Western blot from whole cell lysates to confirm EXT2 knockout in GBM models. β-actin served as loading control. Cropped images are displayed to visualize the protein bands, asterisk indicates EXT2 protein band. Corresponding plating efficiency and clonogenic radiation survival in wild type (WT) and EXT2 knockout (KO) GBM cell models. **F** Western blot from whole cell lysates to evaluate EXT2 overexpression in DD-T4 and U-251MG cells (β-actin and EGFP served as loading and transduction controls, respectively). Cropped images are displayed. Corresponding plating efficiency and clonogenic radiation survival in GBM cell models expressing EGFP empty vector or EGFP/EXT2. Data were normalized to basal conditions (EGFP and 0 Gy). Data in **B** and **C** were statistically analyzed using one-way ANOVA, and in **D,** **E** and **F** were subjected to a two-sided t-test. Results are presented as mean ± SD (*n* = 3; *p < 0.05; **p < 0.01; ***p < 0.005, n.c. not countable).

To explore whether EXT2 generally acts as survival determinant in human GBM cells, we took a heterogeneous panel of human GBM models and silenced EXT2 (Fig. 3A, Supplementary Fig S3A). Intriguingly, we observed significant decreases in cell viability, proliferation, and plating efficiency across the entire cell model panel (Fig. 3B–D, Supplementary Fig. S3B–D). Moreover, 6 out of 8 models demonstrated a significant reduction of clonogenic radiation survival upon EXT2 depletion (Fig. 3D, Supplementary Fig. S3E). According to the applied mathematical model, these reductions turned out to indicate synergism (Supplementary Fig. 3F). This radiosensitization was not due to elevated EXT2 levels upon X-ray exposure (Supplementary Fig. S3G) and did not correlate with basal EXT2 expression levels (Supplementary Fig. S4A, B) or with cell adhesion upon EXT2 depletion (Supplementary Fig. S4C). Consistently, CRISPR/Cas9-mediated EXT2 knockout (KO) in U-251MG and DD-T4 GBM models resulted in decreased basal survival and induced radiosensitization (Fig. 3E, Supplementary Fig. S5), whereas EXT2 overexpression led to increased platting efficiency and radioprotection in both cell models (Fig. 3F).

### EXT2 targeting alters the metabolome of human GBM models

**Fig. 4:**
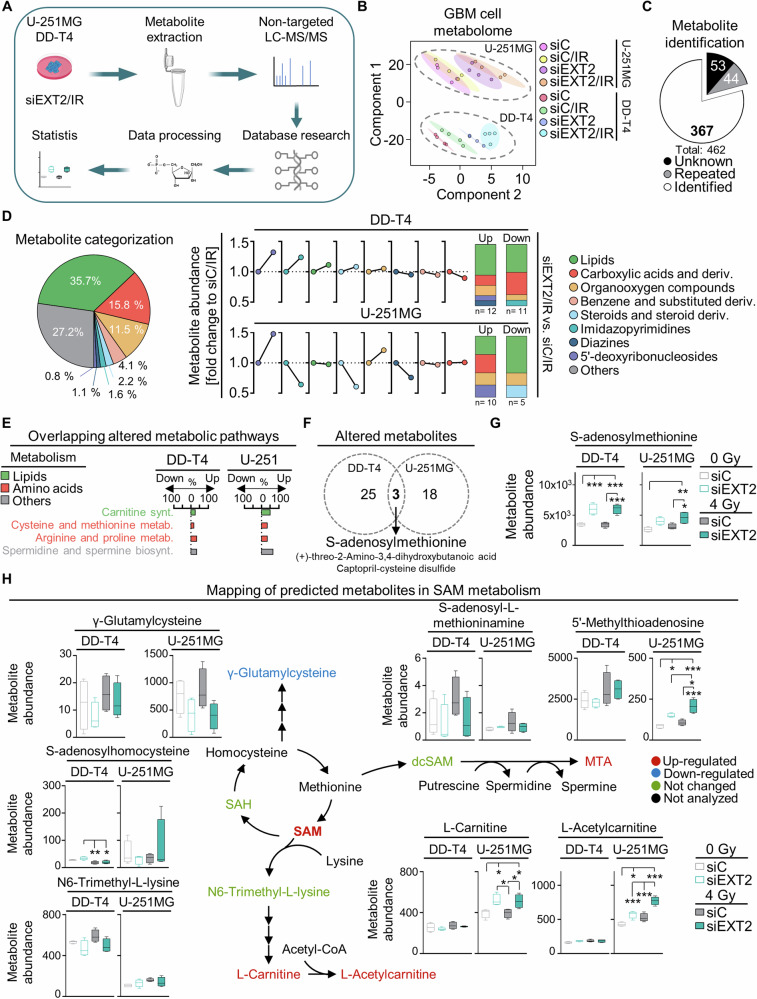
EXT2 targeting alters the metabolome of GBM cells. **A** Workflow of untargeted metabolomics upon EXT2 knockdown in irradiated DD-T4 and U-251MG cells. **B** Differential impact of the treatment groups on the metabolome of DD-T4 and U-251MG GBM models determined by Partial Least-Squares Discriminant Analysis (PLSDA) using MetaboAnalyst.ca web tool. **C** Filtration of LC-MS/MS analytical features for metabolite identification using Human Metabolome Data Base (HMDB) web tool. **D** Categorization of metabolite content by HMDB and determination of changes in metabolite abundance upon EXT2 depletion and irradiation. Numbers in column graph indicate significantly altered metabolites in the respective color-coded metabolite category. Data are visualized as mean (*n* = 4) of the fold change (relative to siC/IR). Significant alterations were determined by *t*-test with *p* < 0.05. **E** Identified deregulated metabolic pathways upon EXT2 knockdown and irradiation obtained from KEGG and Small Molecule Pathway Database (SMPDB) database analyses. The impact is quantified as a percentage of significant up- and down-regulated metabolites. **F** Overlap of significantly altered metabolites, shown in **D** in DD-T4 and U-251MG models. **G** SAM metabolite abundance in unirradiated and irradiated EXT2-depleted cell cultures. **H** Mapping of metabolites involved in SAM metabolism altered upon EXT2 knockdown identified from untargeted metabolomics. Color code: red, upregulated; blue, down-regulated; green, not changed; black, metabolite not found. Metabolite abundance indicates peak intensity value (*n* = 4; one-way ANOVA; **p < 0.01; ***p < 0.005).

Taking the role of EXT2 in the cellular metabolome into consideration, we next explored metabolic modifications by employing non-targeted LC-MS/MS analysis (Fig. 4A). We observed differential and cell model-specific, clustered metabolome changes in response to EXT2 depletion and irradiation (Fig. 4B, Supplementary Fig. S6A). From 462 measured features, 367 potential metabolites could be identified and classified revealing alterations in the metabolite abundance driven by EXT2 depletion and irradiation (Fig. 4C, D, Supplementary Fig. S6B, Supplementary Table S1). The significantly altered metabolites mainly belonged to lipids and amino acids (carboxylic acids and derivatives) (Fig. 4C, D, Supplementary Fig. S6B). Subsequent identification of metabolic pathways in which the significantly up- and downregulated metabolites are active uncovered overlapping effects across four metabolic pathways in both GBM models, namely carnitine synthesis, cysteine and methionine metabolism, arginine and proline metabolism, as well as spermidine and spermine biosynthesis (Fig. 4E, Supplementary Fig. S6C). Comparative analysis between cell models revealed S-adenosylmethionine (SAM) as a key metabolite with increased abundance upon EXT2 depletion in unirradiated and irradiated GBM cells (Fig. 4F, G). Based on feature identification linked to SAM metabolism, additional potential metabolites such as L-Carnitine, L-Acetylcarnitine and 5’-Methylthioadenosine (MTA) (upregulated) and γ-Glutamylcysteine (downregulated) were found altered upon EXT2 targeting in a cell model-dependent pattern (Fig. 4H, Supplementary Table S1).

### EXT2 depletion modulates S-adenosylmethionine metabolism, particularly by modifying the transsulfuration pathway

**Fig. 5:**
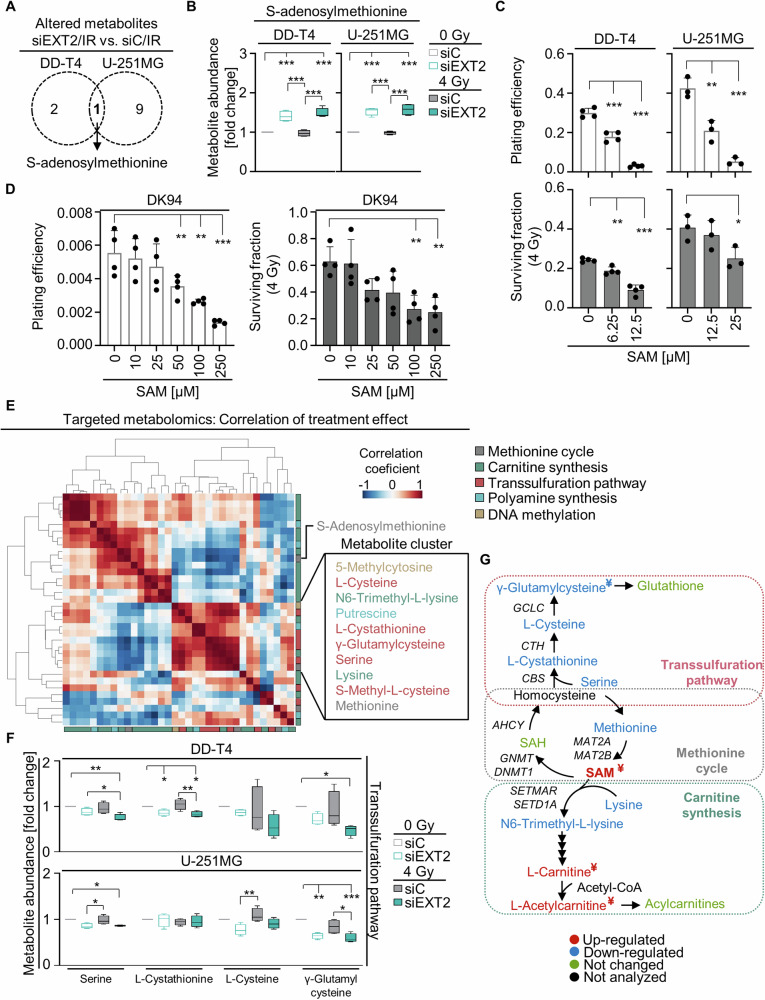
EXT2 depletion deregulates the transsulfuration pathway. **A** Overlap of significantly altered metabolites detected in irradiated EXT2-depleted DD-T4 and U-251MG cell models. **B** SAM metabolite abundance after indicated treatments. Plating efficiency and clonogenic radiation survival of established DD-T4 and U-251MG (**C**) and patient-derived (**D**) GBM cell models treated with indicated concentrations of SAM. Data are shown as mean ± SD (n = 3 in U-251MG (**C**) and n = 4 in DD-T4 (**C**) and **D** and analyzed by one-way ANOVA (*p < 0.05; **p < 0.01; ***p < 0.005). **E** Comparative depiction of changes induced by EXT2 depletion and irradiation in metabolite abundance in DD-T4 and U-251MG cells analyzed by targeted LC/MS-MS. Correlation heatmap was created using Python. Data visualized by Pearson coefficients of the changes in metabolite abundance ordered by hierarchical clustering. **F** Effect of EXT2 knockdown and irradiation on the abundance of indicated transsulfuration pathway metabolites. **G** Mapping of altered metabolites upon EXT2 depletion in methionine cycle, transsulfuration pathway and carnitine synthesis. Color code: red, upregulated; blue, down-regulated; green, not changed; black, metabolite not analyzed. ^**¥**^, altered metabolite found in untargeted metabolomics; SAH: S-Adenosylhomocysteine. Metabolite abundances are normalized to basal conditions (siC, 0 Gy). Data show peak intensity values (*n* = 4; one-way ANOVA; **p < 0.01; ***p < 0.005).

To validate our predictions of altered SAM metabolism gained from untargeted metabolomics, we performed targeted metabolomics encompassing relevant metabolites from the methionine cycle, polyamine biosynthesis, carnitine synthesis, and transsulfuration pathways (Fig. 4H). The increased SAM abundance detected by untargeted metabolomics upon EXT2 knockdown was confirmed by targeted metabolomics (Fig. 5A, B). To prove SAM to mediate cytotoxicity, we applied increasing SAM concentrations and consistently observed reduced cell survival as well as radiosensitization in both GBM cell models and the primary GBM culture DK94 (Fig. 5C, D), thus mirroring the effects mediated by EXT2 depletion.

In a correlation analysis of changes in metabolite levels after EXT2 silencing and irradiation, various metabolite clusters were identified indicating similar alterations in the two GBM models (Fig. 5E, Supplementary Fig. S7A). In our search for metabolites with similarly directed changes in response to EXT2 depletion and irradiation, a metabolite cluster associated with the transsulfuration pathway emerged. Serine, L-Cystathionine, L-Cysteine, and γ-Glutamylcysteine showed, in most cases, significantly decreased abundance in EXT2-depleted and irradiated cells (Fig. 5F). Other metabolites from the investigated pathways confirmed the predictions from untargeted metabolomics, being either non-significantly changed or only altered in one of the two examined GBM models (Fig. 5G, Supplementary Fig. S7A, B).

### EXT2 depletion radiosensitizes GBM cells through activation of ferroptosis

**Fig. 6:**
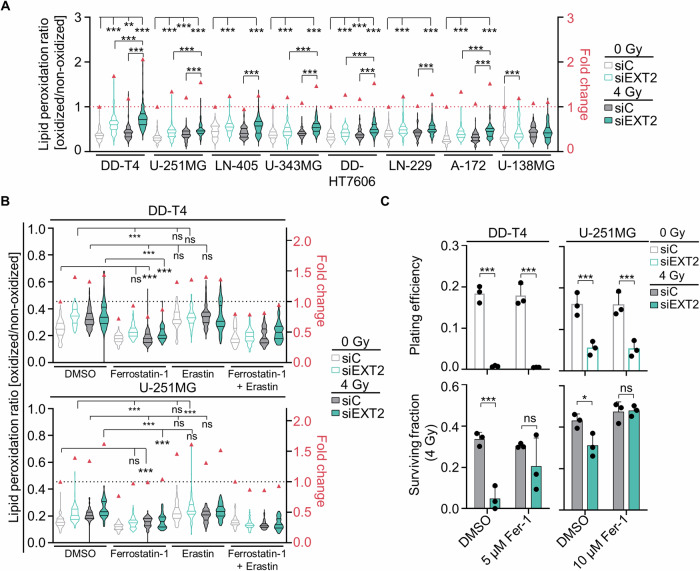
EXT2 inhibition elicits ferroptosis. **A** Lipid peroxidation levels in unirradiated and 4-Gy irradiated EXT2-depleted GBM models. Data were generated from fluorescence images of cells exposed to the oxidized and non-oxidized C11-BODIPY (581/591) sensor. Fifty cells were analyzed per condition using Fiji software. **B** Lipid peroxidation levels in unirradiated and irradiated GBM cells pretreated with either the ferroptosis inducer Erastin or the ferroptosis inhibitor Ferrostatin-1. **C** Plating efficiency and clonogenic radiation survival in Ferrostatin-1-pretreated EXT2-depleted DD-T4 and U-251MG cells. Data show mean ± SD (*n* = 3; one-way ANOVA; *p < 0.05 **p < 0.01; ***p < 0.005).

Given the antioxidant role of the transsulfuration pathway in mitigating ferroptotic cell death, we investigated whether its downregulation through EXT2 depletion is causative for the observed cytotoxicity and radiosensitization via ferroptosis induction. Upon EXT2 silencing, increased lipid peroxidation was evident across the human GBM model panel relative to controls (Fig. 6A). X-ray irradiation further enhanced this effect (Fig. 6A). By means of pretreatment with the ferroptosis inhibitor Ferrostatin-1, the activation of ferroptosis upon EXT2 depletion and irradiation was significantly reversed (Fig. 6B). Pretreatment with the ferroptosis inducer Erastin, in contrast, elevated lipid peroxidation levels without further significant augmentation upon EXT2 silencing or irradiation (Fig. 6B). Importantly, Ferrostatin-1 pretreatment counteracted the radiosensitizing effect of EXT2 silencing in both tested GBM cell models relative to controls, while an effect on basal cytotoxicity was lacking (Fig. 6C, Supplementary Fig. S8).

### EXT2 knockdown induces radiosensitization and ferroptosis by altering SAM and transsulfuration pathway enzymes

**Fig. 7:**
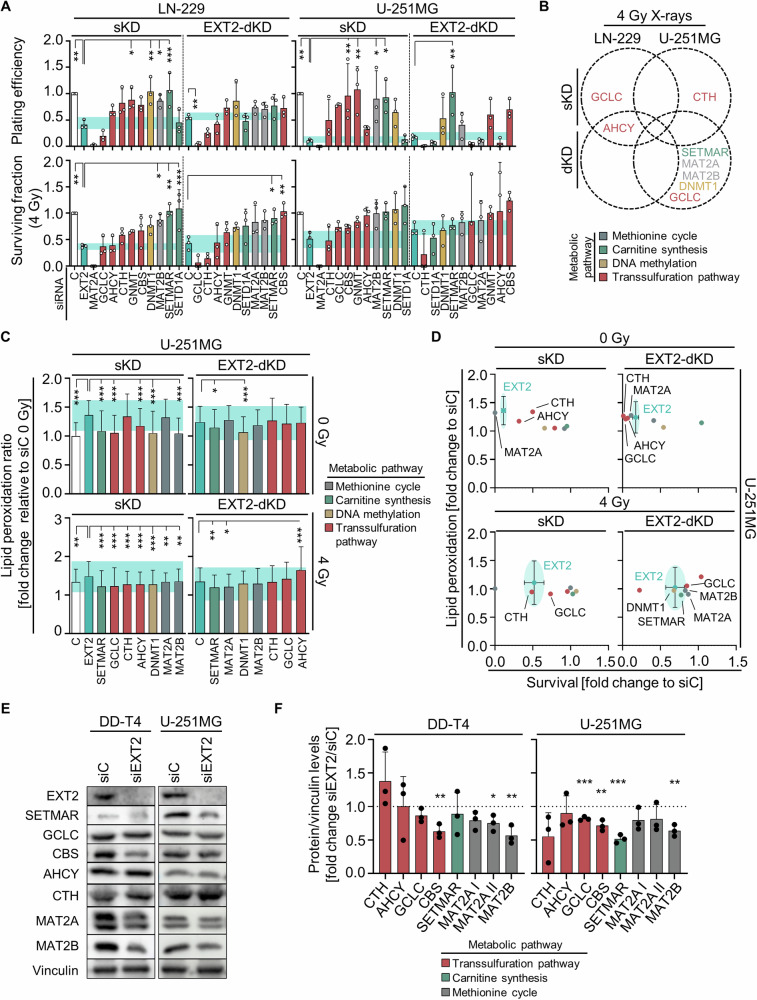
Depletion EXT2 modulates SAM and transsulfuration pathway enzymes. **A** Plating efficiency and clonogenic radiation survival upon single and EXT2-combined double knockdowns of indicated enzymes relative to siC. **B** Venn diagram summarizing the effects measured in **A**. **C** Lipid peroxidation levels in unirradiated and irradiated U-251MG cells upon single and EXT2-combined double depletion of indicated enzymes. **D** Correlation of clonogenic survival and lipid peroxidation as shown in **C** and **A**. **E** Western blotting from whole cell lysates to evaluate changes in the levels of the corresponding enzymes upon EXT2 depletion in DD-T4 and U-251MG GBM models (vinculin served as loading control). Cropped images display representative protein bands. **F** Densitometry analyses from **E**, illustrating the effect of EXT2 knockdown as a fold change values of enzyme/vinculin levels normalized to siC. Data are shown as mean ± SD (*n* = 3) and analyzed by one-way ANOVA (*p < 0.05; **p < 0.01; ***p < 0.005).

We next explored the functional relevance of the transsulfuration and carnitine synthesis pathways by silencing the enzymes involved in SAM synthesis and use, i.e. MAT2A, MAT2B, SETD1A, SETMAR, DNMT1, and GNMT, and in the synthesis of the transsulfuration pathway metabolites such as AHCY, CBS, CTH, and GCLC, without and in combination with EXT2. In general, single and double silencing of these enzymes in combination with EXT2 resulted in a heterogeneous, cell model-dependent response pattern (Fig. 7A). Notably, the basal cytotoxicity observed upon SETD1A and SETD1A/EXT2 depletions mirrored the effects of single EXT2 silencing in LN-229 and U-251MG cells (Supplementary Fig. S9A). In combination with irradiation, this pattern changed uncovering several enzymes with similar radiosensitizing potential as EXT2, in both single as well as in EXT2-combined double depletion. In LN-229 cells, both AHCY and AHCY/EXT2 depletions, as well as GCLC single silencing, exhibited equivalent radiosensitization compared to single EXT2 depletion. Conversely, U251MG cells demonstrated equipotent radiosensitizing effects, similar to EXT2, upon single depletion of CTH and EXT2-double knockdown with SETMAR, GCLC, MAT2A, MAT2B, DNMT1, and GCLC (Fig. 7B). To connect EXT2-induced ferroptosis to the enzymes identified in Fig. 7B, we genetically targeted these enzymes without and in combination with EXT2 and determined lipid peroxidation levels (Fig. 7C, D). Under both non-irradiated and irradiated conditions, the results showed that combined silencing of these enzymes induced similar levels of lipid peroxidation to EXT2 silencing alone (Fig. 7C). The correlation of clonogenic survival and lipid peroxidation revealed that MAT2A alone and combined with EXT2 elicited similar impact on cytotoxicity and lipid peroxidation than EXT2 (Fig. 7D). For irradiation, CTH as well as DNMT1, SETMAR and MAT2A exhibited a similar impact than EXT2 when inhibited alone or in combination with EXT2, respectively (Fig. 7D). We finally assessed changes in the levels of these enzymes in response to EXT2 depletion. Interestingly, we discovered a reduction in the levels of certain enzymes involved in SAM synthesis (MAT2A and MAT2B) and utilization (SETMAR), as well as in the transsulfuration pathway (CBS and GCLC), in the studied GBM models (Fig. 7E, F). Furthermore, a significant correlation at the gene expression level was observed between EXT2 and MAT2B in our cell models as well as in GBM patients (Supplementary Fig. S9B, S10A). However, unlike EXT2, the overexpression of these genes was not associated with worse patient outcomes (Supplementary Fig. S10B, C).

## Discussion

Resistance to therapy remains a hallmark of cancer, often driven by adaptive mechanisms that cancer cells employ to reprogram their metabolism and ensure survival. In recent years, our understanding of these resistance mechanisms has significantly advanced, although many aspects remain unexplored. In this study, we have uncovered a novel role of EXT2 in GBM cell therapy response by revealing that (i) EXT2 depletion reduces cell survival and mediates radiosensitization, (ii) EXT2-depleted GBM cells exhibit dysregulated SAM metabolism, particularly in the downregulation of the transsulfuration pathway, and (iii) ferroptosis plays a pivotal role in the radiosensitizing effect of EXT2 depletion.

Previous studies have established associations between the genetic dysregulation of EXT2 and cancer. In multiple osteochondromas, EXT2 is considered as a tumor suppressor gene, with mutations leading to exostoses development [25]. EXT2 was identified as a prognostic marker in different cancer types such as head and neck squamous cell carcinomas and squamous cell lung carcinoma [26]. In the context of GBM, the function of EXT2 is unstudied and our research reveals that overexpression of EXT2 is linked to shorter patient survival.

In general, EXT2 plays a well-documented metabolic role, as it forms a complex with EXT1, essential for heparan sulfate synthesis [23]. Despite a limited understanding of EXT2 in metabolism, some studies have associated genetic alterations of EXT2 with an increased risk of diabetes [27] and dysregulated nitric oxide metabolism [28]. Furthermore, EXT2 depletion increases lipid deposition, impeding embryonic development [29]. Our investigation in GBM has revealed that EXT2 depletion impacts cell proliferation, cell death, and cell viability. Notably, the role of EXT2 in cancer metabolism has remained largely unexplored. To fill this gap, we conducted comprehensive metabolome analyses, revealing dysregulation in SAM metabolism and associated metabolic pathways.

Elevated levels of SAM were reported to have anti-tumor effects in various cancer types, including GBM, by promoting anti-proliferative effects and inducing different forms of cell death, including apoptosis, autophagy, and ferroptosis [10, 30–32]. Consistent with these findings, our study shows increased levels of SAM following EXT2 depletion, resulting in anti-proliferative effects, similar to those produced by SAM administration, and the induction of different forms of cell death. Altered SAM levels, which act as a methyl donor in various metabolic pathways, have been linked to shifts towards oxidative and lipid metabolism [33, 34]. Indeed, increased SAM levels promote lipid and protein oxidation, and alter the activity of crucial antioxidant enzymes in the brain [35]. Our observations reveal that EXT2 depletion increases the levels of several carnitines in a cell model-dependent manner, which is known to be an antioxidant response to an elevated oxidative environment [36, 37]. We also observed downregulation in the transsulfuration pathway, a well-known antioxidant pathway antagonizing ferroptosis induction [38]. This dysregulation in lipid metabolism and antioxidant capacity may serve as a trigger for lipid peroxidation and explain the activation of ferroptosis after EXT2 depletion. This is in line with studies from others [39–41]. Moreover, it is worth highlighting that ferroptosis is induced in GBM cells upon X-ray irradiation [42, 43]. Our observations uncovered an increased oxidation of lipid peroxides after irradiation, a phenomenon further intensified by the combination of EXT2 depletion. Notably, we observed that the impact of EXT2 depletion on the response to irradiation is, at least partially, mediated by the induction of ferroptosis. This is evident from the fact that pretreatment with the ferroptosis inhibitor, Ferrostatin-1, effectively reverses the radiosensitizing effect upon EXT2 inhibition.

To address the potential underlying molecular mechanism, we performed single and EXT2-combined double knockdown experiments targeting enzymes responsible for the observed metabolite changes. Our experiments suggest a possible connection between EXT2 and metabolic enzymes from various signaling pathways, including the transsulfuration pathway (AHCY, CTH, GCLC), the methionine cycle (MAT2A, MAT2B), carnitine synthesis (SETMAR), and DNA methylation (DNMT1). Moreover, we found dysregulated levels of certain enzymes involved in SAM and transsulfuration pathways (MAT2A, MAT2B, SETMAR, CBS, and GCLC) upon EXT2 depletion, and we identified a significant correlation between EXT2 and MAT2B and AHCY in both GBM cell models and patients at the gene expression level. Intriguingly, single knockdowns of these enzymes did not elicit the same effects as EXT2 knockdown alone, probably due to the activation of compensatory mechanisms [44, 45], suggesting that the effect of EXT2 downregulation on cytotoxicity and lipid peroxidation may be mediated by a combined participation of dysregulated enzymes. Therefore, EXT2 may directly or indirectly regulate SAM utilization and feedback inhibition, with EXT2 depletion leading to increased SAM levels and further dysregulation of these metabolically active enzymes. In accordance, increased SAM levels have been reported to produce negative feedback and decrease the levels of MAT2A [46], CBS [47], and MAT2B [48]. The connection between EXT2 and these enzymes is further supported by our observations that EXT2/CBS double knockdown reverses the cytotoxicity of EXT2 silencing, while EXT2/MAT2A double knockdown partially reverses the cytotoxicity of MAT2A depletion. This also suggests that, although the levels of these enzymes are partially reduced by EXT2 KD, the specific metabolic conditions in the absence of EXT2 play a major role. Although no direct link has been found between dysregulated SAM levels and the other enzymes, the regulation of SAM levels seems to be controlled by the activities of these enzymes, implying an interconnected feedback system accounting for the shared effects upon their depletion. Due to its complexity, further investigations are warranted to untangle the hierarchical interrelations between the here presented enzymes. However, the translational potential of EXT2 targeting in its connection to SAM is high based on the known functions of SAM in both cancer and normal cells, possibly due to its role as a major methyl donor and epigenetic regulator [49]. Elevated SAM induces both anti- and pro-cell death effects in normal and cancer cells, respectively [32, 50, 51]. In the future, in-depth comparative studies on the differential existence of SAM itself and interacting enzymes in cancer cells compared to normal cells are required. This may open a therapeutic window for effective and safe application of SAM or specific targeting of its associated enzymes.

In summary, our study demonstrates that EXT2 depletion impairs GBM cell survival and overcomes radioresistance through dysregulation of SAM metabolism and ferroptosis. This study enhances our understanding of the adaptive reprogramming strategies GBM cells employ to survive and resist therapy. Future investigations are needed for clarifying the role of EXT2 as a potential therapeutic target.

## Materials and methods

### Cell lines

U-87MG, A-172, LN-229 and U-138MG GBM cell lines were obtained from American Type Culture Collection (ATCC Manassas, Virginia, USA). DD-T4, DD-HT7606 and U-343MG GBM cells were kindly provided by A. Temme (University Hospital Dresden, Germany). LN-405 cells were purchased from the Leibniz Institute DSMZ and U-251MG cells from the Cell Lines Service (CLS) GmbH (Eppelheim, Germany). GBM cells were cultured in Dulbecco’s modified Eagle’s medium (DMEM, Thermo Fisher Scientific, Waltham, MA, USA) supplemented with 10% fetal bovine serum (FBS, PAN-Biotech, Aidenbach, Germany) and 1% non-essential amino acids (Sigma-Aldrich, Taufkirchen, Germany.) at 37 °C and 8.5% CO_2_. U-343MG cells grow in Basal Medium Eagle (BME, Thermo Fisher Scientific), supplemented with 10% FBS, 10 mM 4-(2-hydroxyethyl)-1-piperazineethanesulfonic acid (HEPES), 2 mM L-glutamine, and 1% non-essential amino acids (all from Sigma-Aldrich) maintained at 37 °C and 5% CO_2_. HEK293T cells were cultured in DMEM supplemented with 10% FBS and 1% penicillin/streptomycin (Sigma-Aldrich). All cell lines were regularly tested negative for mycoplasma contamination and authenticated using STR DNA profiling.

### Primary GBM cell cultures

Human DK94 primary GBM cultures were generated as described [52] and cultured in neurobasal medium (Life Technologies, Carlsbad, CA, USA) supplemented with 2 mM L-Glutamine (Life Technologies), 32 U/mL Heparin (Sigma-Aldrich), B27 supplement (Life Technologies), 20 ng/mL human EGF (R&D Systems, Wiesbaden-Nordenstadt, Germany) and 20 ng/mL human FGFb (Life Technologies). Cultures were maintained at 37 °C and 5% CO_2_ for no more than 5 passages after thawing. Regular testing ensured cells were free from mycoplasma contamination. Cells were authenticated using STR DNA profiling.

### Antibodies

Primary antibodies were purchased as indicated: LC3B (D11) XP® (#3868; Cell Signaling Technology, Frankfurt a. M., Germany), γH2AX (05-636; Millipore, Burlington, MA, USA), 53BP1 (NB100-304; Novus Biologicals, Littleton, Colorado, USA), EXT2 (#PA5-26408; Invitrogen, Waltham, MA, USA), β-actin (#4970S; Cell Signaling Technology). MAT2A (#55309-1-AP, Proteintech, Planegg-Martinsried, Germany), MAT2B (#15952-1-AP, Proteintech), AHCY (#10757-2-AP, Proteintech), CTH (#12217-1-AP, Proteintech), CBS (#14787-1-AP, Proteintech), GCLC (#12601-1-AP, Proteintech), SETMAR (#25814-1-AP, Proteintech). Secondary antibodies: AlexaFluor488 anti-mouse (A11029); AlexaFluor546 anti-rabbit (A11010) were acquired from Life Technologies; EasyBlot anti-Mouse IgG (HRP), (#GTX221667-01); and EasyBlot anti-Rabbit IgG (HRP) (#GTX221666-01) were purchased from GeneTex (Irvine, CA, USA).

### RNA isolation and sequencing

RNA was isolated using the RNeasy Mini Kit (Qiagen, Venlo, The Netherlands) according to manufacturer’s instructions. RNA integrity number was determined using the 4200 Tape Station (Agilent Technologies, Santa Clara, California, USA). Samples of four biological replicates of each GBM cell model were submitted for RNA sequencing to the DRESDEN-concept Genome Center (Technische Universität Dresden, Germany). mRNA was isolated from 400 ng DNase treated total RNA using NEBNext rRNA depletion Kit (New England Biolabs, Ipswich, Massachusetts, USA) according to the manufacturer’s instructions. Samples were directly subjected to the workflow for strand-specific RNA-Seq library preparation (NEBNext Ultra II Directional RNA Library Prep, NEB). For ligation custom adaptors were used (Adaptor-Oligo 1: 5’-ACA CTC TTT CCC TAC ACG ACG CTC TTC CGA TCT-3’, Adaptor-Oligo 2: 5’-P-GAT CGG AAG AGC ACA CGT CTG AAC TCC AGT CAC-3’). After ligation, adapters were depleted by a XP bead purification (Beckman Coulter, Brea, California, USA) adding beads in a ratio of 1:0.9. Dual indexing was done during the following PCR enrichment (15 cycles, 65 °C) using custom amplification primers carrying the index sequence indicated with ‘NNNNNNN’. (Primer1: AAT GAT ACG GCG ACC ACC GAG ATC TAC ACT CTT TCC CTA CAC GAC GCT CTT CCG ATC T, primer2: CAA GCA GAA GAC GGC ATA CGA GAT NNNNNNNN GTG ACT GGA GTT CAG ACG TGT GCT CTT CCG ATC T). After two more XP bead purifications (1:0.9) libraries were quantified using the Fragment Analyzer (Agilent Technologies). Libraries were equimolarly pooled before sequencing them 2 ×100 bp paired-end on a NovaSeq 6000 system to a depth of on average 60 million read pairs per sample. The quality of RNA-seq raw reads was checked by FastQC v0.11.4 and Trim Galore v0.4.2 was used for adapter trimming, followed by the alignment of raw reads to the human reference genome (GRCh38 Ensembl release 95) by using STAR v2.5.3a with the standard settings [53]. Duplicated raw reads were marked using Picard tools v1.141 and the quality of the genome mapped reads was determined by analyzing read distributions across gene bodies with RSeQC v3.0.02. Raw read counts per gene were determined by counting gene-specific reads in exons of protein-coding genes using FeatureCounts v1.5.3 [54]. Finally, a gene expression data matrix was created by removing genes without any reads and lowly expressed genes (less than 1 read per million in more than 50% of samples) followed by cyclic loess normalization [55] resulting in normalized log-2 counts per million. A genome-wide gene expression heatmap was created in R (heatmap.2) and cell line samples were clustered based on their gene expression profile using one minus Pearson correlation as distance measure in combination with Ward’s clustering method (ward.D2). To identify genes that were strongly expressed across all cell lines, the t-test was used to determine if the average log-expression level of a gene across the cell lines was significantly greater than zero. Correction for multiple testing was done by computing FDR-adjusted p-values [56]. Detailed information can be found in Supplementary Table S2 for RNA-seq raw data as counts per million (CPM) and Supplementary Table S3 for strongly expressed genes.

### Small interfering RNA knockdown

ON-TARGETplus siRNA library was purchased from Dharmacon™-Horizon Discovery (Cambridge, UK). Specific siRNA sequences targeting: GNS, HAT1, VDAC1, CAPZA1, ANXA2, GNG12, HSPA5, EXT2, GOSR2, B4GALT7, PDIA3, HSP90B1, MAT2A, MAT2B, SETD1A, SETMAR, DNMT1, GNMT, AHCY, CBS, CTH, GCLC and a non-targeting control are listed in Supplementary Table S4. For siRNA transfection, GBM cells were seeded 18 h prior to incubation with Opti-MEM (Thermo Fisher Scientific) with 9.1 nM of the siRNA and 5 µL of lipofectamin RNAiMAX reagent (Invitrogen) for 24 h, as published [57]. In the screen, knockdown efficacies were not individually checked. For each siRNA transfection, three biological replicates were evaluated.

### Treatments of cells

GBM cells were treated with the following pharmacological agents: Temozolomide (MSD Sharp & Dohme, Haar, Niedersachsen, Germany), Bafilomycin A1 (BVT-0252; Biomol, Hamburg, Germany), Ferrostatin-1 (Cay17729; Biomol), Erastin (Cay17754; Biomol), and S-adenosylmethionine (SAM) (B9003S; New England Biolabs) using the indicated concentration and time.

### Radiation exposure

Cells were irradiated at room temperature with the selected dose point of 4 Gy of 200 kV X-rays filtered with 0.5 mm Cu (Yxlon Y.TU 320; Yxlon, Hamburg, Germany) as published [58]. The dose rate during irradiation was approximately 1.3 Gy/min at 20 mA. The absorbed dose was measured using a Duplex dosimeter (PTW, Freiburg, Germany).

### Cell viability

Cell viability was determined by measuring the activity of metabolic enzymes using Cell Titer-Blue reagent (Promega, Walldorf, Germany). Briefly, a cell line-dependent number of cells was seeded in black 96-well plates (Corning, Corning, NY, USA) in three technical replicates and cultured for 24 h prior to treatment administration. Four days later, cell viability was evaluated by adding 20 μL of the reagent and incubation in darkness for 4 h. Fluorescence was recorded at 560_Ex_/590_Em_ using the Cytation 5 Cell Imaging Multi-Mode Reader (Agilent Technologies). For each condition, three biological replicates were evaluated.

### Apoptosis analysis

To assess apoptosis, a cell line-dependent number of cells was seeded in black 96-well plates (Corning) in three technical replicates and treated 24 h later. Following 24 h of treatment, caspase 3/7 activity was measured by adding Apo-ONE® Assay reagent (Promega) in a 1:1 ratio and incubation in darkness at room temperature for 2 h. Fluorescence of three biological replicates per condition was measured at 485_Ex_/530_Em_ using the Cytation 5 Cell Imaging Multi-Mode Reader (Agilent Technologies).

### Foci assay

Residual DNA DSBs were detected by the presence of foci (labeled 53BP1 and γH2AX) as published [57]. Briefly, GBM cells were seeded on glass cover slips and treated 24 h later. After treatment, cells were fixed and permeabilized with 3% formaldehyde/PBS and 0.25% Triton-X-100/PBS. 1% BSA/PBS was used as a blocking solution and for diluting antibodies. Coverslips were placed on glass object slides with 10 μL of ProLong™ Diamond Antifade Mountant with DAPI (Thermo Fisher Scientific). Dry coverslips were imaged using Axio Imager 2 (Carl Zeiss Microscopy GmbH, Jena, Germany) fluorescent microscope. The number of foci was counted in 50 cells in each condition of the three biological replicates using Fiji software and representative images were taken with 20X magnification.

### LC3 immunostaining

To assess autophagy, GBM cells on cover slips were exposed to treatment along with the autophagy flux inhibitor Bafilomycin A1 (10 nM) as published [59]. On the next day, cells were fixed using 100% MetOH for 15 min at -20 °C. 1% BSA/PBS was used as blocking buffer and for diluting LC3B and secondary antibody. Coverslips were placed on glass object slides with 10 μL of ProLong™ Diamond Antifade Mountant with DAPI (Thermo Fisher Scientific). The Axio Imager 2 (Carl Zeiss Microscopy GmbH) was used for counting LC3B puncta numbers in 50 cells in each condition of the three biological replicates using Fiji software and for taking representative images with 20X magnification.

### Generation of EXT2 knockout and EXT2 overexpression cell models

The generation of EXT2 knockout in U-251MG and DD-T4 GBM cell models was conducted as previously described [60]. EXT2-specific gRNA1 (5’-CACCGAAAGCCGCCACCAGCCAACA-3’), gRNA2 (5’-CACCGAGATCCACCTCAGCTGACAG-3’) and gRNA3 (5’-CACCGATCTTCCAGAGAAAGGACCA-3’) were designed using the Synthego CRISPR design tool (https://www.synthego.com/products/bioinformatics/crispr-design-tool) and cloned into pL.CRISPR-puro vector. gRNA efficiency was estimated by PCR amplification of the site around the cut (forward primer 5’-GGATAGAACGCAGCTGATGG-3’, reverse primer 5’-TAGAGGAGGAGGATTCGGGG-3’), followed by bulk PCR Sanger sequence analysis performed using Synthego ICE analysis tool (https://ice.editco.bio/#/) to determine indel frequency and cutting score. For overexpression of EXT2 in U-251MG and DD-T4, human EXT2 was cloned into pL.OE as previously published [61]. HEK293T cells were transfected with the lentiviral plasmids containing either the gRNAs or EXT2, psPAX2 (#12260, Addgene, Watertown, MA, USA) and pMD2.G (#12259, Addgene), both generously provided by D. Trono. Two days later, the supernatant containing the lentiviral particles was collected, ultracentrifuged and used for infection of U-251MG and DD-T4 cells. EXT2 knockout and EXT2 overexpression were confirmed by western blot analysis.

### Total protein extraction and western blotting

Forty-eight hours after transfection, GBM cells were lysed with 1x RIPA lysis buffer (50 mM Tris HCl (pH 7.4, AppliChem, Darmstadt, Germany), 150 mM NaCl (Roth, Karlsruhe, Germany), 1% Nonidet-P40, 0.25% sodium deoxycholate, 1 mM EDTA, 1 mM NaVO4, 2 mM NaF (all Sigma-Aldrich) and Complete™ Protease Inhibitor Cocktail (Roche, Basel, Switzerland)) as published [59]. Protein concentration measurements were performed using BCA assay kit (Thermo Fisher Scientific). Following SDS-PAGE, proteins were transferred onto nitrocellulose membranes (GE Healthcare, Chicago, IL, USA) and detected by incubating the membranes with specific primary antibodies and horseradish peroxidase-conjugated secondary antibodies. Protein bands were detected with an ECL Prime Western Blotting Detection Reagent (GE Healthcare) using a Fusion FX (Vilber Lourmat GmbH, Eberhardzell, Germany). Protein bands were normalized to β-actin or vinculin. For each condition, three biological replicates were evaluated.

### Colony formation assay

To determine the clonogenicity, single cells were seeded in either 24-well or 6-well plates as published [59]. After 24 h, the cells were irradiated with 4 Gy of X-rays and subsequently cultured for a cell line-specific duration until visible colonies were formed. The colonies were fixed using 80% EtOH and stained with Coomassie blue dye (Merck Millipore, Burlington, MA, USA). Colonies consisting of more than 50 cells were counted using a stereomicroscope (Carl Zeiss Microscopy GmbH). For each condition, three technical replicates were evaluated within each of the three or four biological replicates.

### Cell count assessment of cell proliferation

Cell proliferation was evaluated by subculturing 1000 cells/well in a 24-well plate format 24 h after siRNA transfection. The following day, cells were irradiated and allowed to grow. On the sixth day, cells from the three technical replicates within each of the three biological replicates were detached and manually counted using a Neubauer chamber.

### Adhesion assay

To assess the adhesion capacity, 40,000 cells were seeded per well on collagen type I-coated (1 µg/cm^2^, BD Biosciences, San Jose, California, USA) 24-well plates. After 30 min, non-attached GBM cells were removed by washing twice with complete medium. The attached cells were then fixed using 80% EtOH and stained with Coomassie blue dye. Adhered cells were scanned using the Cytation 5 Cell Imaging Multi-Mode Reader (Agilent Technologies) and counted using Fiji software. For each condition, three biological replicates were evaluated.

### Gliomasphere formation assay

The reproductive integrity of primary GBM cells was assessed by measuring their ability to form spheres after treatment. In this experiment, 6000 DK94 cells were seeded in low-attachment 96-well plates in NBM with SAM or DMSO as a control. After 2 h, cells were irradiated with 4 Gy X-rays or left unirradiated and cultured under normal culture conditions. After 6 days, spheres were fixed with formaldehyde, and spheres larger than 100 μm were counted using a microscope.

### Metabolomics sample preparation

Forty-eight hours after siRNA transfection, cells growing in 10 cm dishes were irradiated with 4 Gy X-rays. One hour after irradiation, cells were washed twice in PBS, followed by metabolite extraction using 1 mL of −80 °C cold quenching buffer (80/20 MeOH/H2O; Optima™ LC/MS (Fisher Bioreagents, Pittsburgh, Pennsylvania, USA)/Molecular Biology Grade H_2_0 (Fisher Bioreagents) and centrifugation. Sample extract supernatants from four biological replicates were dried by vacuum-assisted centrifugation.

### Non-targeted metabolomics

Metabolite profiling based on liquid chromatography high-resolution tandem mass spectrometry (LC-HR-MS/MS) was performed using an untargeted metabolomics approach. The instrument set-up contained an ultra-performance liquid chromatography system (Aquity I-class; Waters GmbH, Eschborn, Germany) coupled to a quadrupole-time-of-flight mass spectrometer (QToF) additionally equipped with an ion mobility spectrometer (IMS) (VION IMS QToF; Waters GmbH, Eschborn, Germany). For analysis, sample extract residue was reconstituted in 200 µL aqueous acetonitrile (10% water) including 0.1% formic acid and transferred into autosampler glass vials. A pool containing 20 µL from each of those samples was prepared in a separate autosampler vial and analyzed multiple times randomly throughout the analysis. Chromatographic separation was achieved by applying hydrophilic interaction chromatography (HILIC) using a BEH Amide column (2.1 × 100 mm, 1.7 µm; Waters GmbH, Eschborn, Germany) at 45 °C in conjunction with a gradient of mobile phase A (water including 0.1% formic acid) and mobile phase B (acetonitrile including 0.1% formic acid). After sample injection starting with 1%/99% A/B at a flow rate of 0.4 mL/min, mobile phase A was linearly increased to 5% within 2 min, followed by a further increase to 99% reached at 7.5 min. After another 0.5 min, starting conditions were re-established within 0.3 min followed by column equilibration at starting conditions for 1.7 min. Positive and negative electrospray ionization (ESI) in combination with a high-definition data acquisition scan mode (HDMS^E^) that included ion mobility screening in conjunction with determination of metabolite-specific collision cross section (CCS) values, and accurate mass as well as respective fragment ion mass screenings. The observed mass range included mass-to-charge ratios between 50 and 1000 Da. Total scan time was set to 0.3 s from which 40% of the time a collision energy of 6 eV (low energy) was applied before collision energy was respectively ramped either from 15 to 50 eV or 10 to 80 eV (high energy) in positive or negative ESI mode for the remaining 60% of the time. Ion source parameters in positive ESI mode were respectively set to 1 kV and 40 V for capillary voltage and sample cone voltage and to 120 °C and 550 °C for source and desolvation temperature. In negative ESI mode, parameters were set to 1.5 kV and 40 V, and to 120 °C and 450 °C, respectively. Nitrogen was applied as cone gas and desolvation gas with flow rates of 50 L/h and 1000 L/h, respectively, in positive and negative ESI. Repeated injections of a Leucine-Enkephalin solution served as reference for mass correction. Data acquisition and raw data processing was performed by using the Waters Unifi software package 2.1.2. For further data processing, Unifi export files derived from positive and negative ESI screenings were imported into the Progenesis QI software (Nonlinear Dynamics, Newcastle upon Tyne, UK). Processing included peak picking with auto threshold and chromatographic alignment using an automatically chosen reference sample, signal deconvolution and data normalization as well as set-up of experimental design. For metabolite identification, features derived from untargeted metabolomics screenings were compared with the publicly available human metabolome database (HMDB, www.hmdb.ca) using the Progenesis Metascope plugin applying a precursor mass accuracy of ≤10 ppm and a theoretical fragment mass accuracy of ≤10 ppm. Furthermore, features were compared to in-house data achieved by injections of metabolite standards in a neat solution including allowed deviations of retention time and CCS of 0.3 min and 3%, respectively. Metabolic features that have not received a metabolite annotation were excluded, remaining features in conjunction with their signal intensities and respective identifications were exported to Microsoft Excel for further data processing.

### Targeted metabolomics

Targeted metabolite profiling was done by LC-MS/MS using an instrument set-up containing an ultra-performance liquid chromatography system (Aquity I-class; Waters GmbH) coupled to a triple quadrupole linear ion trap mass spectrometer (QTRAP 6500 + ; Sciex, Darmstadt, Germany). Chromatographic separation was achieved by using an Acquity BEH Amide column (100 mm × 2.1 mm, 1.7 µm; Waters GmbH) at 40°C in conjunction with a gradient of an aqueous mobile phase A (5% methanol, 0.2% formic acid) and a mobile phase B acetonitrile/methanol (95%/5%, 0.2% formic acid). Five µL of reconstituted test samples, kept at 6 °C in the autosampler were injected into the LC-MS/MS system at a flow rate of 0.4 mL/min with 30% mobile phase A. At 0.37 min proportions of mobile phase A increased linearly to 60% at 1.4 min, and further to 95% at 2 min. After a hold until 3.5 min, the gradient returned back to initial conditions at 3.8 min and was then kept for another 2.7 min for column equilibration. Metabolites were detected in multiple reaction monitoring scan mode (MRM) using positive electrospray ionization (ESI). Optimization of compound-dependent source and fragmentation parameters was done by single standard injections, standards derived from an available mass spectrometry metabolite library (MSMLS; Sigma-Aldrich Chemie GmbH, Taufkirchen, Germany), using the instrument-integrated syringe pump (Supplementary Table S5). A flow injection analysis was used to further optimize the ionization source parameters with curtain gas (35 psi), ESI voltage (4500 V), source temperature (500 °C), gas 1 (70 psi) and gas 2 (60 psi). Data acquisition was performed by Analyst 1.7 (Sciex). Data processing was done by using Sciex OS-MQ software package. Data assessment regards fold changes was achieved by using respective analyte peak areas of target metabolites.

### Lipid peroxidation assay

Seventy-two hours after siRNA transfection and/or twenty-four hours after treatment with X-ray or inhibitors, cells seeded in 35 mm glass bottom Petri dishes were incubated with 2 µM Bodipy 581/591 lipid peroxidation sensor (Thermo Fisher Scientific) diluted in Hanks’ Balanced Salt solution (HBSS, Thermo Fisher Scientific) for 30 min at 37 °C. Upon fixation using 4% formaldehyde/PBS, cells were imaged using Axio Observer Z1 (Carl Zeiss Microscopy GmbH; 20X magnification) and analyzed by measuring the ratio of oxidized (510_EM_) and non-oxidized (590_EM_) lipid peroxides using Fiji software. For each condition, three biological replicates were analyzed.

### Public database analysis

Patient data was obtained from TCGA GBM dataset using different web tools. DEG in GBM patients relative to normal tissue were obtained from GEPIA, Oncomine and XENA browser. We employed Betastasis for Kaplan-Meier survival analysis to correlate gene expression with patient outcomes and gsea-msigdb for functional analysis. For the metabolome analysis, the Human Metabolome Data Base (HMDB) identified and categorized the metabolites. Kyoto Encyclopedia of Genes and Genomes (KEGG) and Small Molecule Pathway Database (SMPD) were used to classify the metabolites into their corresponding metabolic pathways.

### Statistical analysis

The centered value is defined as the mean ± standard deviation (SD), used to calculate and plot error bars of at least three independent biological experiments (indicated as n) is shown. We did not pre-specify the effect size for sample calculations. For determining statistical significance, two-sided Student’s *t*-test or one-way ANOVA, followed by post-hoc analysis using Tukey’s or Dunnet´s methods, were employed using Prism8 (GraphPad) or Microsoft Excel 2016. For gene expression analysis, *t*-test was used to determine if the average log-expression level across the tested cell models was significantly greater than zero compared to the human reference genome. Pearson correlation was used for determining the degree of correlation using Python. For GBM patient survival, log-rank test statistical analysis was performed using Python. *p*-value of less than 0.05 was considered statistically significant and indicated as *p < 0.05; **p < 0.01; ***p < 0.005. Treatment synergism was assessed by the Bliss definition of drug independence [62]. Non-targeted metabolomics data processing and statistics were performed on the Metaboanalyst platform (MetaboAnalyst.ca web tool). Normalized metabolite peak intensities (relative to siC 0 Gy) were transformed to ensure normal distribution and significantly altered metabolites were identified by *t*-test.

## Supplementary information

Supplemental Information

Supplementary Table S1

Supplementary Table S2

Supplementary Table S3

Supplementary Table S4

Supplementary Table S5

Original western blots

## Data Availability

All data will be made available to the scientific community upon request.
