# Supplementary material for: A novel role of exostosin glycosyltransferase 2 (EXT2) in glioblastoma cell metabolism, radiosensitivity and ferroptosis
Source: Cell Death Differ. 2025 Apr 15;32(9):1664–78. doi: 10.1038/s41418-025-01503-w (PMC12432244; doi:10.1038/s41418-025-01503-w)
Supplement: Supplementary file 1 — Supplemental Information [file 41418_2025_1503_MOESM1_ESM.pdf]

Supplemental Information

A novel role of exostosin glycosyltransferase 2 (EXT2) in glioblastoma cell metabolism, radiosensitivity and ferroptosis

Rocío Matesanz-Sánchez, Mirko Peitzsch, Inga Lange, Jovan Mircetic, Michael Seifert, Nils Cordes, and Anne Vehlow

Supplementary Figures

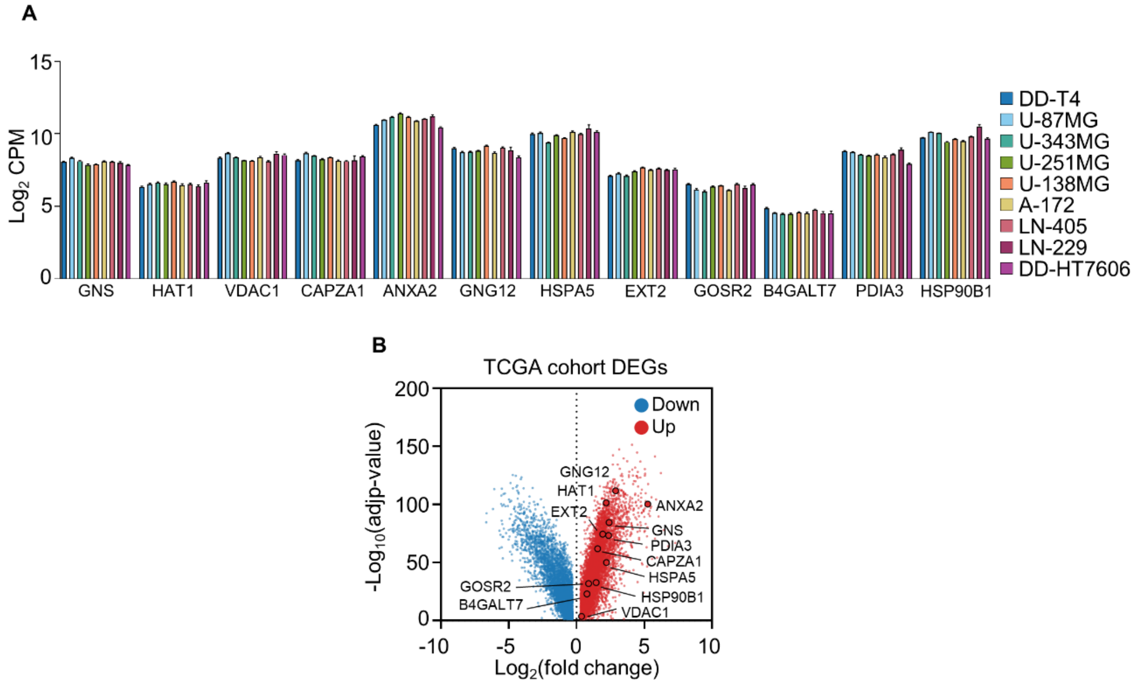

Supplementary Fig. S1. mRNA expression of identified gene candidates in tested GBM models. (A) Candidate gene expression in GBM cell models. RNA-seq data are shown as mean ± SD (n = 4) of the log$_2$ counts per million (CMP). **(B)** Visualization of candidate DEG from TCGA GBM patient cohorts in a Volcano plot. Significantly up- and downregulated genes are shown in red and blue, respectively, and determined by ANOVA with a cut-off $|Log_2$ (fold change GBM versus normal brain)$| > 0.3$, $p < 0.05$, using GEPIA web tool.

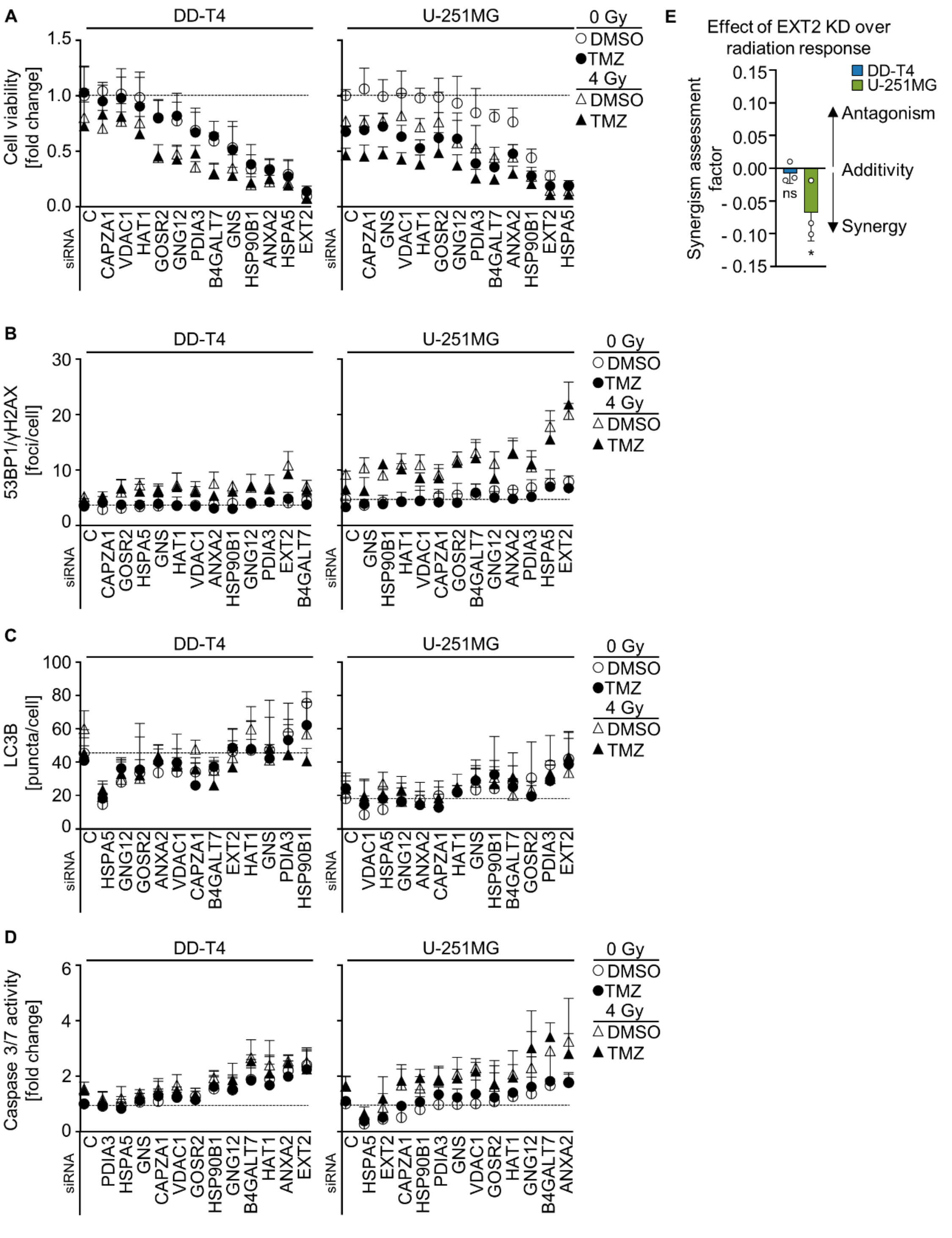

Supplementary Fig. S2. Identification of EXT2 as the most promising candidate mediating the radiation response of human GBM models. Determination of **(A)** cell viability, **(B)** residual DSB (53BP1/γH2AX foci), **(C)** autophagy (LC3B puncta), and **(D)** apoptosis (Caspase 3/7 activity) in GBM models depleted of indicated candidate genes upon TMZ/irradiation. Data are shown as mean ± SD (n = 3) of the values normalized to the basal conditions (siC DMSO/0 Gy)

for cell viability and apoptosis and non-normalized data for autophagy and DSB. For 53BP1/γH2AX foci (**B**) and LC3B puncta (**C**), 50 cells were analysed per condition using the Fiji software. (**E**) Evaluation of synergism for reduced cell viability upon EXT2 depletion plus irradiation (Data from Fig. 2B, Supplementary Fig. S2A). The synergism assessment factor indicates antagonism when values > 1, additivity for values $= 1$, and synergy for values < 1. Synergy was statistically determined using the Bliss independence model (*$p < 0.05$; ns = not significant).

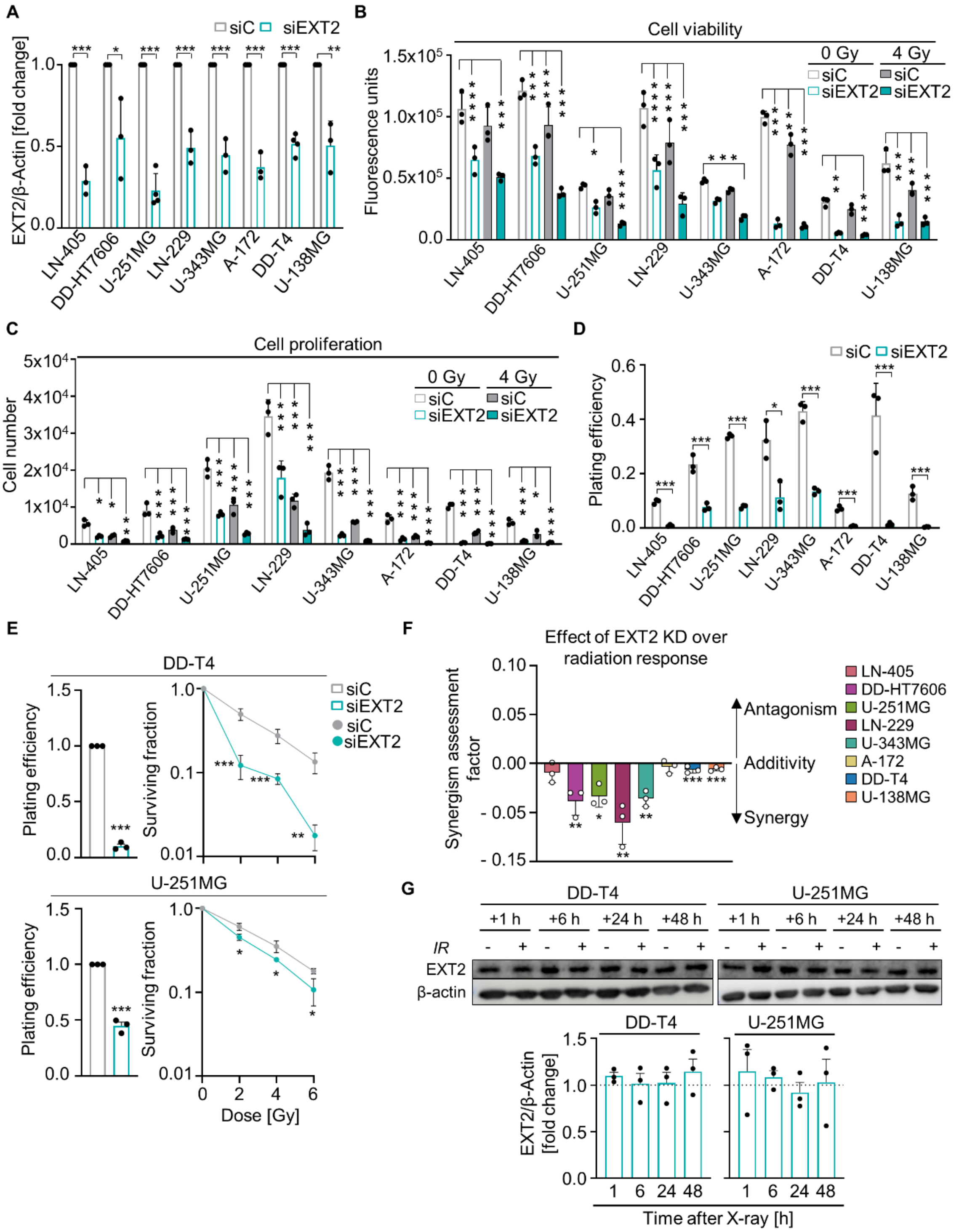

Supplementary Fig. S3. EXT2 silencing reduces cell viability, proliferation, plating efficiency and clonogenic radiation survival. (A) Densitometry from whole cell lysates for quantification of EXT2 knockdown efficiency (β-actin served as loading control). **(B)** Cell viability (fluorescence units), **(C)** cell proliferation, and **(D)** plating efficiency of indicated GBM models after EXT2 depletion and irradiation relative to controls. **(E)** Effect of EXT2 knockdown

on plating efficiency and clonogenic radiation survival at different X-ray doses in DD-T4 and U-251MG cells. **(F)** Evaluation of synergistic effects on GBM clonogenicity after combined EXT2 knockdown and irradiation (data from **Fig. 3D**). Synergism Assessment Factor indicates when values > 1 antagonism, =1 additive, and < 1 a synergy. The statistical determination of synergy was performed using the Bliss independence model. **(G)** Western blot and densitometry analyses of whole cell lysates to assess EXT2 levels after X-ray irradiation in DD-T4 and U-251 cells. β-actin served as the loading control. Cropped images are displayed to visualize the protein bands. Protein quantification was performed using the Fiji software. Data are presented as mean ± SD (n = 3) and compared with either one-way ANOVA (**A**, **B**, **C**) or t-test (**D**, **E**) (*p < 0.05; **p < 0.01; ***p < 0.005).

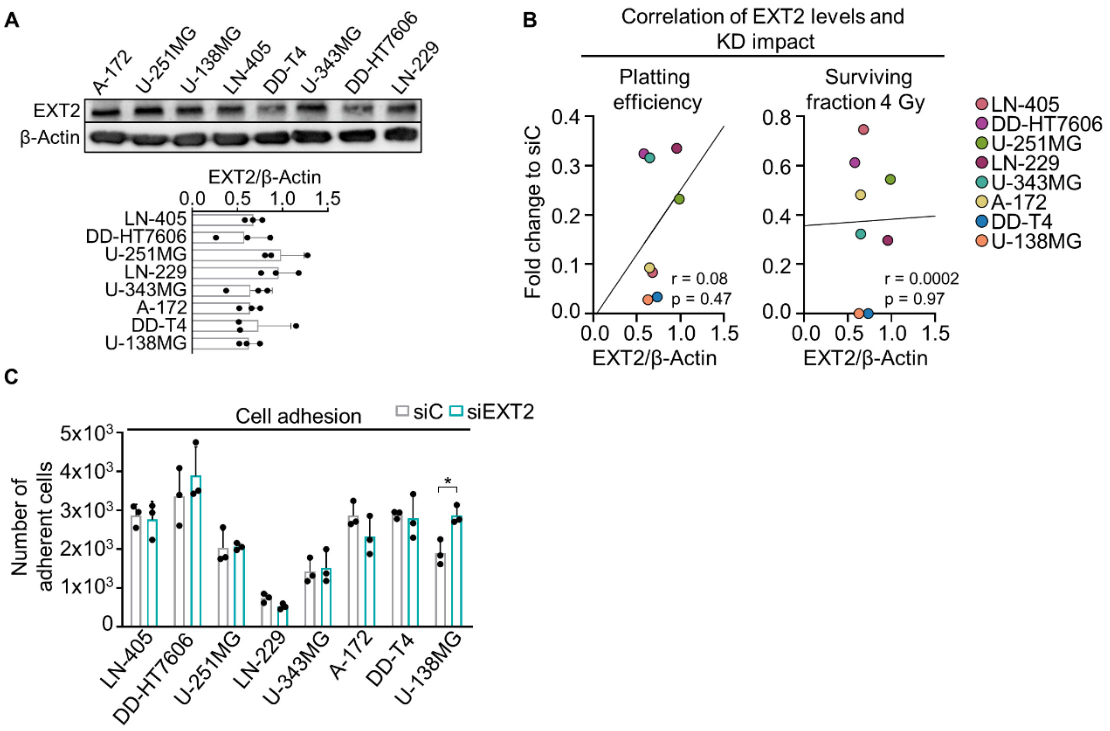

Supplementary Fig. S4. EXT2 silencing reduced survival does not correlate with basal EXT2 levels or changes in cell adhesion. (A) Western blotting and densitometry from whole cell lysates for quantification of basal EXT2 expression (β-actin used as loading control). Cropped images are displayed. **(B)** Correlation of basal EXT2 expression levels and either plating efficiency or clonogenic radiation survival of EXT2-depleted cells. Data are normalized to siC. The linear regression curve, Pearson's correlation score (r) and p-value are indicated. **(C)** Cell adhesion of EXT2-depleted GBM cells. Number of attached cells was determined using the Fiji software. Data are presented as mean ± SD (n = 3) and compared with t-test (*p < 0.05).

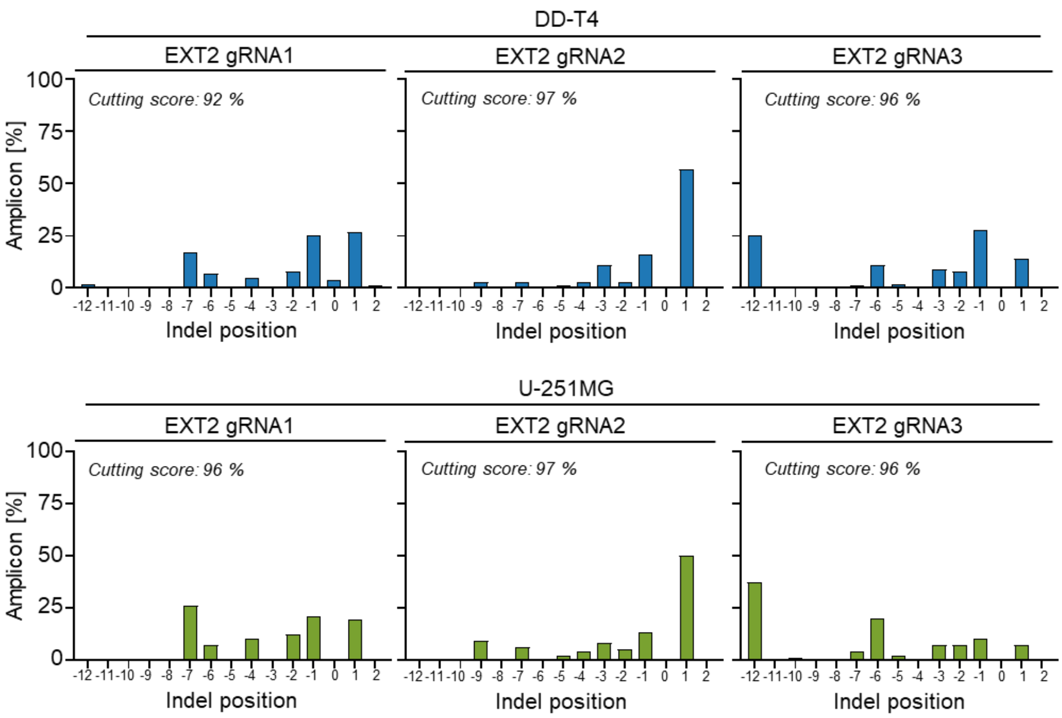

Supplementary Fig. S5. EXT2 depletion in combination with X-ray irradiation in EXT2 knockout GBM cells. On-target efficiencies of all gRNA used in DD-T4 and U-251MG cells. The plots depict frequency of indels of indicated size.

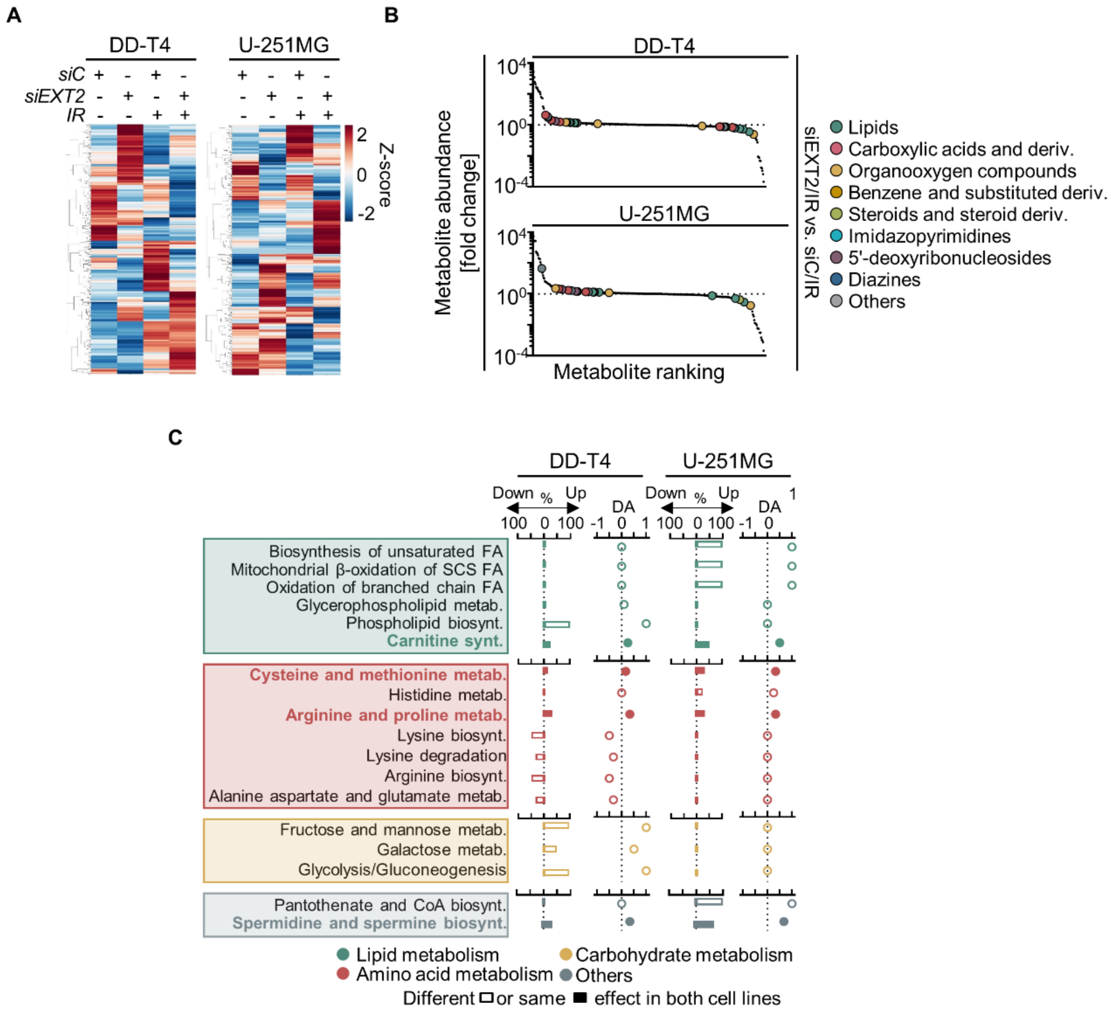

Supplementary Fig. S6. Depletion of EXT2 modulates the GBM cell metabolome. (A) Heatmap of metabolome alterations upon EXT2 knockdown (siEXT2) and X-ray irradiation (IR) created in Python and metabolite peak intensities are displayed in rows as a mean (n = 4) in Z-scored scale and clustered hierarchically. **(B)** Altered metabolites upon EXT2 depletion in irradiated cells. Data are visualized as mean (n = 4) of the fold change (relative to siC/IR) of metabolite abundance. Significant alterations (t-test; $p < 0.05$) are indicated in the corresponding metabolite category colors. **(C)** Identified deregulated metabolic pathways upon EXT2 knockdown after irradiation obtained from KEGG and SMPDB database analyses. The impact is quantified as percentage of significantly up- and down-regulated metabolites and the differential abundance (DA), indicating the direction of change.

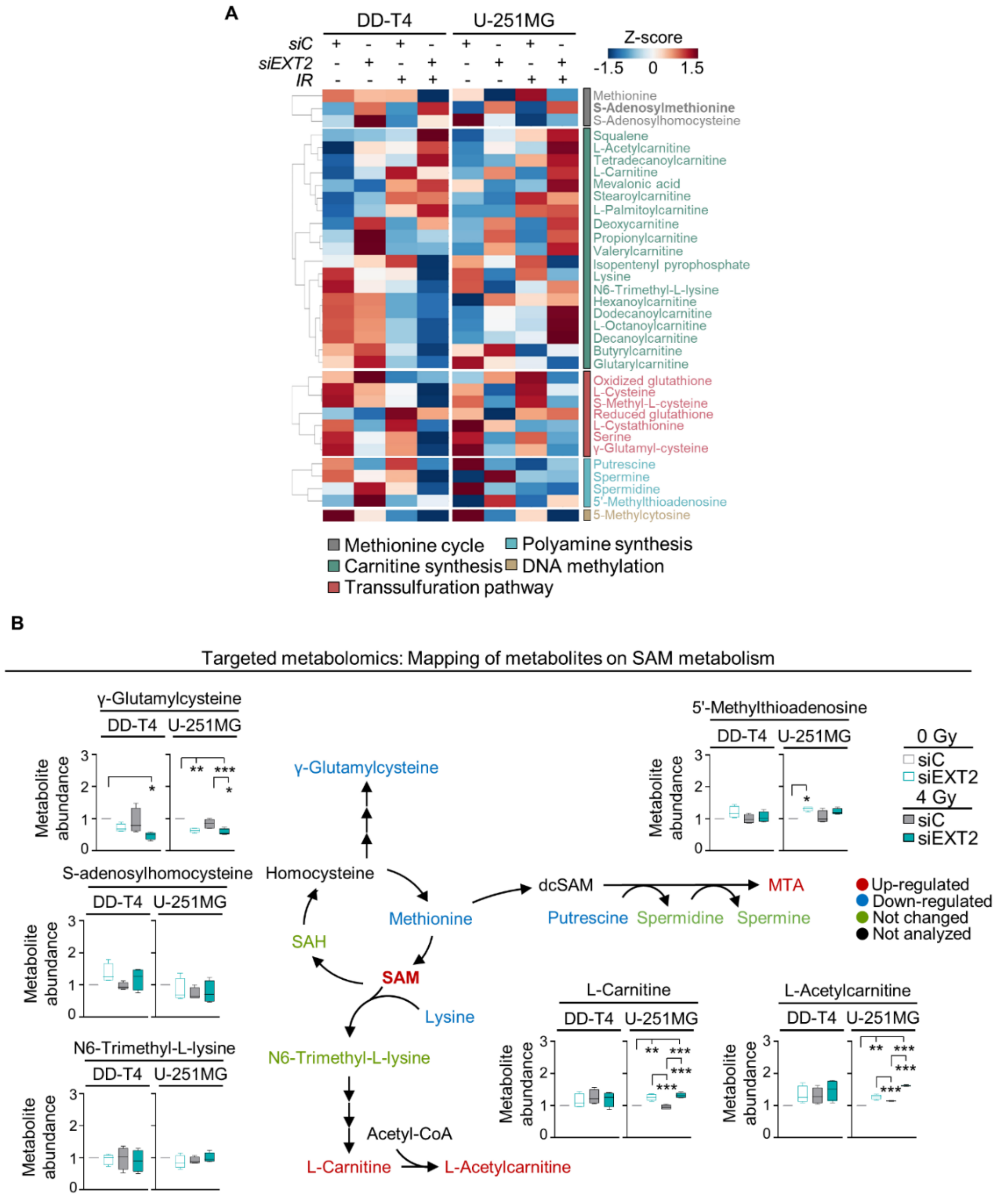

Supplementary Fig. S7. EXT2 depletion impacts on SAM metabolism. (A) Differential abundance of studied metabolites after EXT2 depletion and irradiation in DD-T4 and U-251MG cells, analyzed by targeted LC-MS/MS. Heatmap (created in Python) shows the metabolite peak intensities in z-scored scale, presented as a mean (n = 4), and ordered by hierarchical clustering in DD-T4 cells. **(B)** Mapping of indicated metabolites from SAM metabolism analyzed by targeted metabolomics. The impact of EXT2 knockdown and irradiation on metabolite levels are

expressed as fold change of the peak intensity (relative to basal siC) and analyzed by performing one-way ANOVA ($n = 4$; *$p < 0.05$; **$p < 0.01$; ***$p < 0.005$).

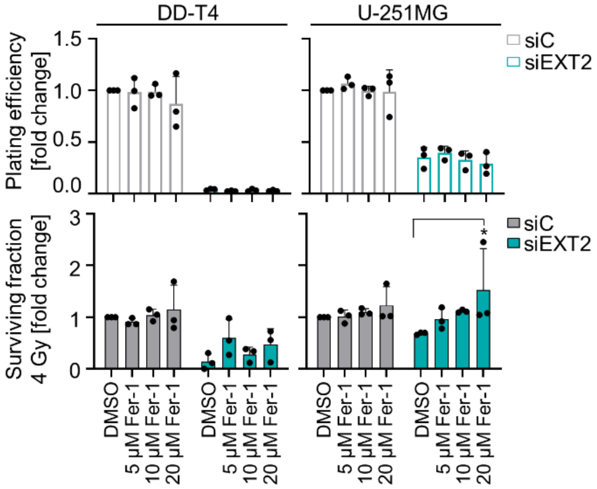

87

88 **Supplementary Fig. S8. EXT2 silencing induces ferroptosis.** Effect of increasing

89 concentrations of Ferrostatin-1 on plating efficiency and clonogenic radiation survival of EXT2-

90 depleted DD-T4 and U-251MG cells. Data show mean ± SD (one-way ANOVA; *p < 0.05).

91

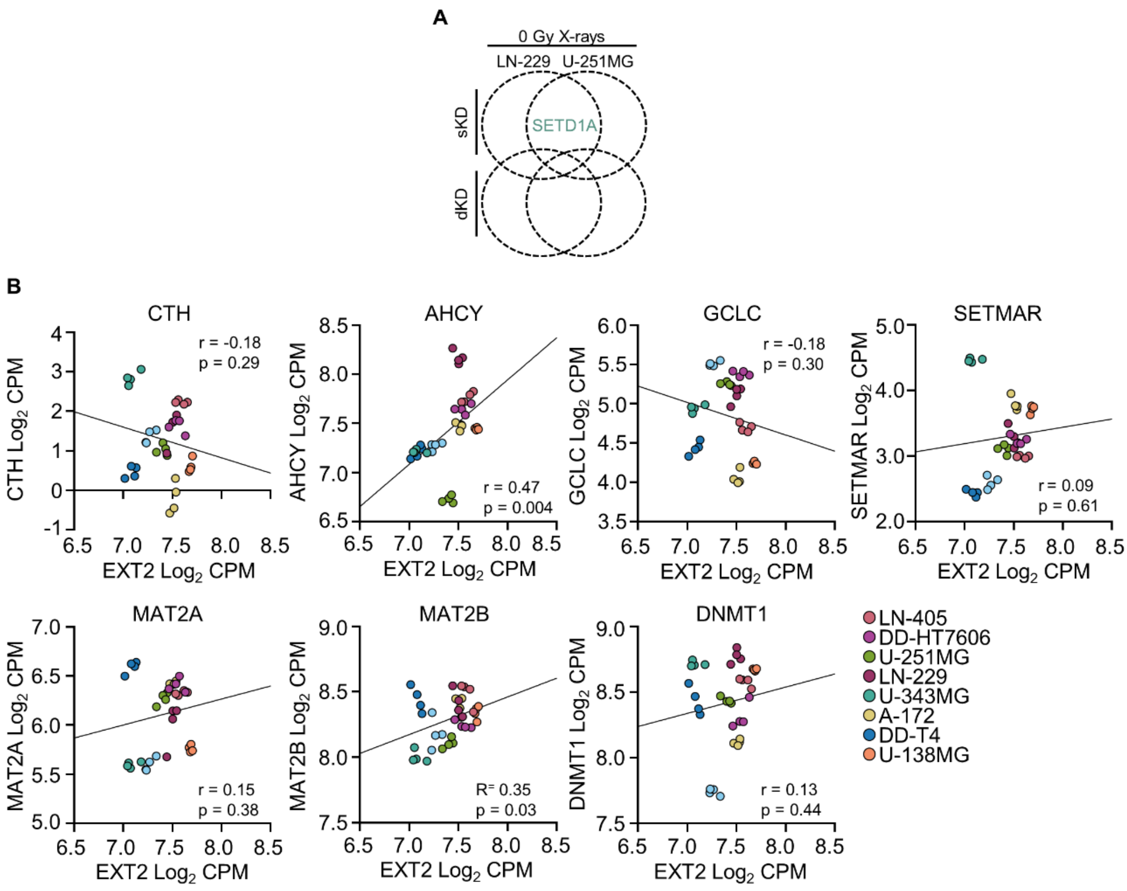

Supplementary Fig. S9. Targeting EXT2 is associated with changes in SAM-related and transsulfuration pathway enzymes in GBM cells. (A) Venn diagram depicting the enzymes that exhibited overlapping effects in cytotoxicity upon either single or EXT2-combined double depletion. **(B)** Correlation of EXT2 and SAM-related enzyme gene expression in GBM cell models. RNA-seq data are shown as mean ± SD (n = 4) of the Log_2 counts per million (CMP). Correlation is indicated by Pearson correlation coefficient (r) and p-value.

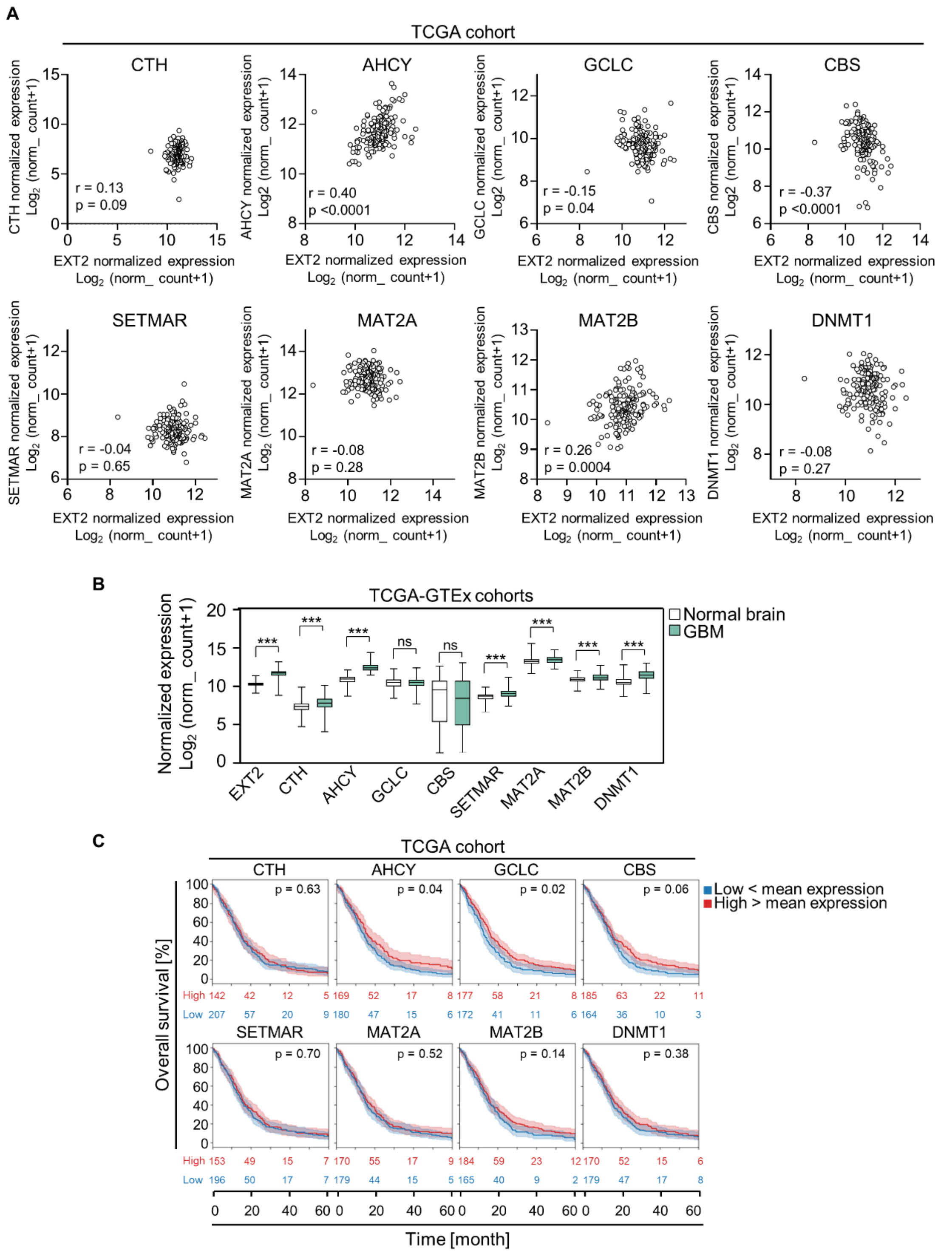

Supplementary Fig. S10. SAM-related and transsulfuration pathway enzyme expressions correlate with EXT2 without associating with survival in GBM patients. (A) Correlation of EXT2 and SAM-related enzyme gene expression in GBM patients. Clinical data shown as normalized gene expression expressed with Log$_2$ (norm_count+1) generated by the TCGA Research Network (https://www.cancer.gov/tcga and acquired from Xena

(https://xenabrowser.net/). Correlation is indicated by Pearson correlation coefficient (r) and p-value. **(B)** Comparative mRNA expression in tumor and normal brain tissue. Clinical data shown as normalized gene expression expressed as Log_2 (norm_count+1) generated by the TCGA Research Network (https://www.cancer.gov/tcga and acquired from Xena (https://xenabrowser.net/). Statistical significance was determined by one-way ANOVA (***p < 0.005, ns = not significant). **(C)** Overall survival analyses based on gene expression (low versus high) from the TCGA GBM patient cohorts. Kaplan-Meier curves display the confidence intervals, the long-rank test p-values and the patient numbers. Data were downloaded from betastasis.com.

Supplementary Table S4. Specific siRNA sequences targeting the indicated genes.

Gene	Sequences
GNS	UGUCAGGACUCGAAGAUUU, CCAAGACUCCAAUGACUAA, GAACGAACAAGCACUGGUU, UGACUUGGGUCCUACUAUU
HAT1	CAAUGAAGAUAUGGCGAUA, GCACAAACACGAAUGAUUU, GAAUAUGCAUCUAAAGUUG, GAAGAUUACCGGCGUGUUA
VDAC1	UAACACGCGCUUCGGAAUA, GAAACCAAGUACAGAUGGA, GAGUACGGCCUGACGUUUA, CCUGAUAGGUUUAGGAUAC
CAPZA1	CACUAACUGUUUCGAAUGA, GACGUUCGGCUACUACUUA, GAUGGGCAACAGACUAUUA, UCUGUACUGUUUAUGCUAA
ANXA2	CGACGAGGACUCUCUCAUU, AUCCAAGUGUCGCUAUUUA, AAAACCAGCUUGCGAAUAA, GGAAGAAAGCUCUGGGACU
GNG12	GAAUAAAGGUUUCGAAGGC, UUAGAAGCCUCCAUUGAAA, GGAAUACCAACUUCAGAAA, CAGCAAGCACCAACAAUAU
HSPA5	GCGCAUUGAUACUAGAAAU, GAACCAUCCCGUGGCAUAA, GAAAGAAGGUUACCCAUGC, AGAUGAAGCUGUAGCGUAU
EXT2	GUGCGUCGGUCAAGUAUAA, GGUCAAACAUGGAGAGUCA, CGAAUCACCUGUUGUUCAA, GGUCAGAGUGCAUCAACAA
GOSR2	ACGAAUCACUGCAGUUUAA, CGAAAUCCAAGCAAGCAUA, GAUCCAGUCUUGCAUGGGA, UUAGAUGGGCACAAUAUUU
B4GALT7	GGGCAGCGCUCAUCAACGU, GCAAUGGGAUGUCCAACCG, CAACUGGGUACAAGACAUU, CAACAGCACGGACUACAUU
PDIA3	GGAAUAGUCCCAUUAGCAA, GGGCAAGGACUUACUUAUU, AGACCCAAAUAUCGUCAUA, GAGGAGUUCUCGCGUGAUG
HSP90B1	GAAGAUGGCCAGUCAACUU, GCGAUUACAUUAAGCUCUA, GGACGGGGAACGACAAUUA, UAGAAGAGGAUCUGGGUAA
MAT2A	GAAAGUGGUUCGUGAAGCU, GUGUUCAUCUUGACAGAAA, GACCAGGGCUUAAUGUUUG, GAAAGGAUUAUACCAAGGU
MAT2B	GAGCACAACGUCCGAGAAA, GGGCAUUGGCCAACGAACA, UGUAAGGACCAAACUUCUA, GACAAACGGUCUUUCAUUA
DNMT1	GCACCUCAUUUGCCGAAUA, AUAAAUGAAUGGUGGAUCA, CCUGAGCCCUACCGAAUUG, GGACGACCCUGACCUCAAA
GNMT	ACAAGUGGGUCAUCGAAGA, CAAGCAGCCUUCGGAGGUA, GGAUGACUCUGGACAAAGA, GAACAUCUACUAUAAGAGU
SETMAR	CAAGUGUUCAAGACGCAUA, CCGUAGAAAAGUCGAACAU, CUUGGAAUUUAUACCGAAA, UGACAACCGGCGACGAUCA
SETD1A	CGGAAAGAGCCAUCGGAAA, CUCAGAAGGUGUACCGCUA, AGUAUAUACCAGUCGAAGA, ACAUAUGUUAAUAGCGCAA
AHCY	GACCCAUCCAGACAAGUA, GCGAAACGGACGAGGAGUA, CAAGGUCCUCCAUCAAU, GCAUUGAGCAGACCCUGUA
CBS	AGACGGAGCAGACAACCUA, ACAGGACGGUGGUGGACAA, GUGCGGAACUACAUGACCA, GGAAGAAGUUCGCCUGAA
CTH	GUACAGGGAUGGUCACCUU, GCACUCGGGUUUUGAAUAU, CUACAUGUCCGAAUGGAAA, GAGCUUGGGAGGAUUCGAA
GCLC	GCGAUGAGGUGGAAUACAU, UAACAGACUUUGAGAACUC, UGGCAGACAAUGAGAUUUA, CCAUCUCCAUUUAUAGAAA
ON-TARGETplus Non-targeting Control	UGGUUUACAUGUCGACUAA, UGGUUUACAUGUUGUGUGA, UGGUUUACAUGUUUUCUGA, UGGUUUACAUGUUUUCCUA
