# Supplementary material for: A novel role of exostosin glycosyltransferase 2 (EXT2) in glioblastoma cell metabolism, radiosensitivity and ferroptosis
Source: Cell Death Differ. 2025 Apr 15;32(9):1664–78. doi: 10.1038/s41418-025-01503-w (PMC12432244; doi:10.1038/s41418-025-01503-w)

EXT2 KD validation panel GBM cell models (Fig. 3A)

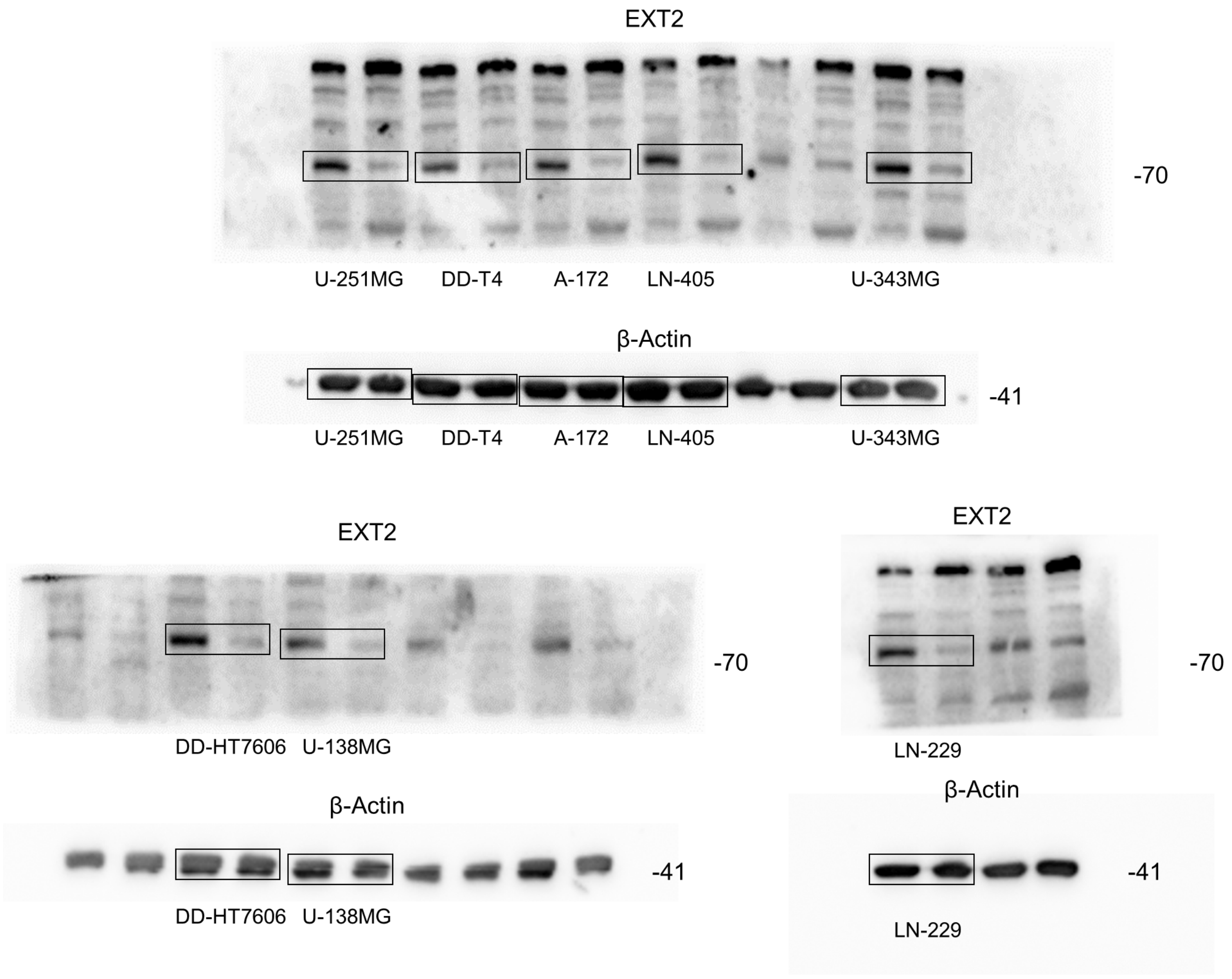

EXT2 levels after X-ray (Supplementary Fig. S3G)

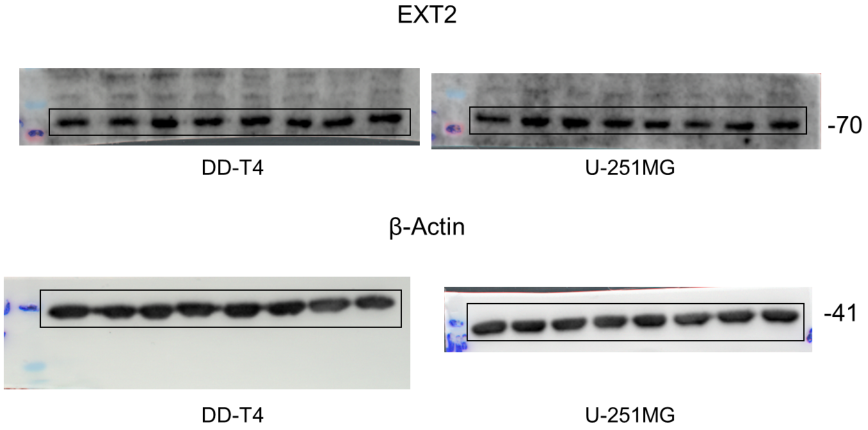

EXT2 Knockouts (Fig. 3E)

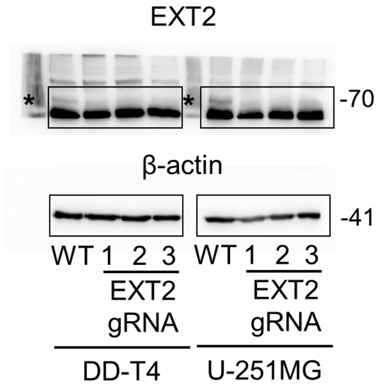

EXT2 overexpression (Fig. 3F)

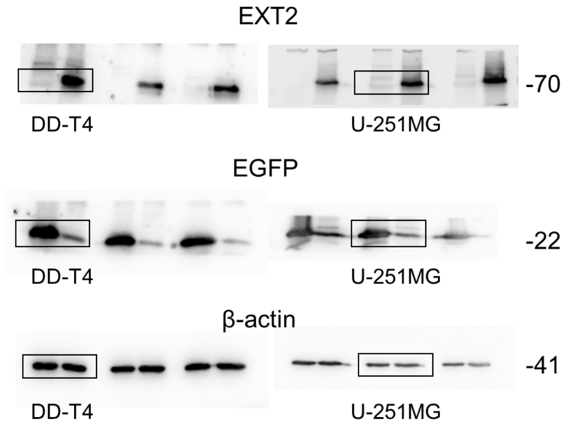

EXT2 expression levels (Supplementary Fig. S4A)

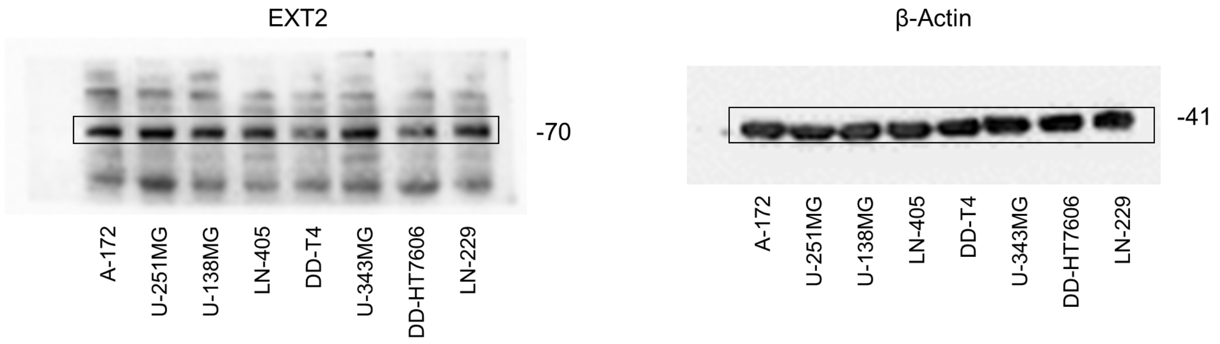

Enzyme levels upon EXT2 KD (Fig. 7E)

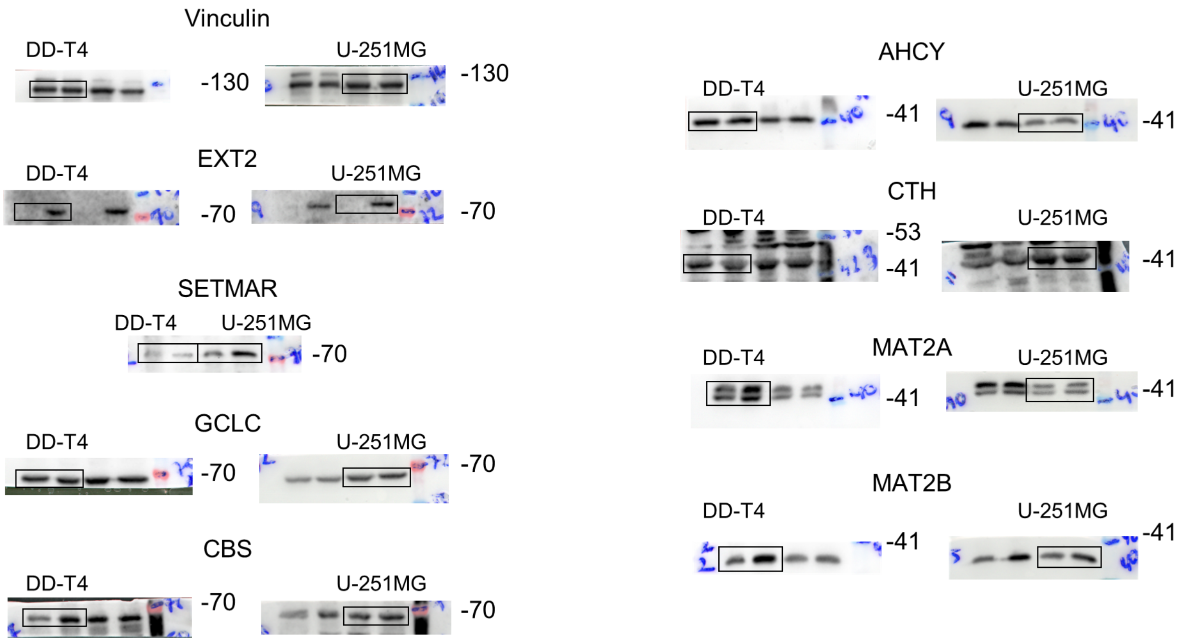

Supplement: Supplementary file 7 — Original western blots [file 41418_2025_1503_MOESM7_ESM.pdf]
